# Synthesis and Characterization of Novel Amphiphilic *N*-Benzyl 1,4-Dihydropyridine Derivatives—Evaluation of Lipid Monolayer and Self-Assembling Properties

**DOI:** 10.3390/ma16124206

**Published:** 2023-06-06

**Authors:** Anna Krapivina, Davis Lacis, Martins Rucins, Mara Plotniece, Karlis Pajuste, Arkadij Sobolev, Aiva Plotniece

**Affiliations:** 1 Department of Pharmaceutical Chemistry, Faculty of Pharmacy, Riga Stradiņš University, Konsula 21, LV-1007 Riga, Latvia; akrapivina17@gmail.com (A.K.); mara.plotniece@rsu.lv (M.P.); 2 Latvian Institute of Organic Synthesis, Aizkraukles 21, LV-1006 Riga, Latvia; davis.lacis@osi.lv (D.L.); rucins@osi.lv (M.R.); kpajuste@osi.lv (K.P.); arkady@osi.lv (A.S.); 3 Faculty of Materials Science and Applied Chemistry, Riga Technical University, P. Valdena 3/7, LV-1048 Riga, Latvia

**Keywords:** *N*-benzyl 1,4-dihydropyridines, pyridinium amphiphiles, langmuir monolayer, mean molecular area, self-assembling, nanoparticles, lipoplexes, DLS

## Abstract

Liposomes and other nanoparticles have been widely studied as innovative nanomaterials because of their unique properties. Pyridinium salts, on the basis of 1,4-dihydropyridine (1,4-DHP) core, have gained significant attention due to their self-assembling properties and DNA delivery activity. This study aimed to synthesize and characterize original *N*-benzyl substituted 1,4-dihydropyridines and evaluate the influence on structure modifications on compound physicochemical and self-assembling properties. Studies of monolayers composed of 1,4-DHP amphiphiles revealed that the mean molecular areas values were dependent on the compound structure. Therefore, the introduction of *N*-benzyl substituent to the 1,4-DHP ring enlarged the mean molecular area by almost half. All nanoparticle samples obtained by ethanol injection method possessed positive surface charge and average diameter of 395–2570 nm. The structure of the cationic head-group affects the size of the formed nanoparticles. The diameter of lipoplexes formed by 1,4-DHP amphiphiles and mRNA at nitrogen/phosphate (N/P) charge ratios of 1, 2, and 5 were in the range of 139–2959 nm and were related to the structure of compound and N/P charge ratio. The preliminary results indicated that more prospective combination are the lipoplexes formed by pyridinium moieties containing *N*-unsubstituted 1,4-DHP amphiphile **1** and pyridinium or substituted pyridinium moieties containing *N*-benzyl 1,4-DHP amphiphiles **5a**–**c** at N/P charge ratio of 5, which would be good candidates for potential application in gene therapy.

## 1. Introduction

The application of nanotechnology from material science to healthcare has led to the development of a variety of novel nanoproducts which have substantially improved the disease diagnosis, monitoring, and treatment [1]. Various basic nanomaterial forms have been tested in the field of drug delivery and diverse therapies. The small size of nanoparticles as delivery systems allows them to cross biological barriers and target tumors, tissues, and individual cells [2].

Due to their unique properties, liposomes and other nanoparticles have been widely studied as innovative nanomaterials for the transmembrane delivery of a wide range of carriers (DNA, RNA, drugs, magnetic dots, etc.) [3,4]. Liposomes are composed of a lipid bilayer with an outside aqueous core, and each of them can be employed for the transport of drugs in the treatment of disease [5]. When compared to the traditional drug delivery systems, liposomes exhibit good biocompatibility, dual character (ability to entrap either hydrophobic or hydrophilic drugs) [6], an ability to protect the encapsulated substances from physiological degradation [7], extend the half-life of the drug, and control the release of drug molecules with lower toxic side effects [8]. All this reveals the potential of liposomes as prospective delivery systems. Thus, numerous liposomal drug formulations have been approved and have started to be used in clinics [9], mainly for cancer treatment [10]. Recently, the successful application of liposomal delivery systems was verified in two COVID-19 messenger RNA (mRNA) vaccines from Pfizer/BioNTech and Moderna [11].

Various delivery system physicochemical attributes, such as size, surface chemistry, shape, and mechanical flexibility, influence their therapeutic futures, i.e., particle uptake and uptake mechanism, blood circulation time, and biodistribution [12]. The physicochemical properties of parent natural or synthetic lipids forming these nanocarriers highly influence the resulting properties of delivery systems. Thus, variations of substituents, and their lipophilicity, steric, and electronic nature, could significantly influence the properties of delivery systems, forming monomers and final nanoaggregates—size, shape, and surface charge (zeta-potential)—with the following effect on particle–cell interactions and biodistribution [13,14,15].

**Figure 1:**
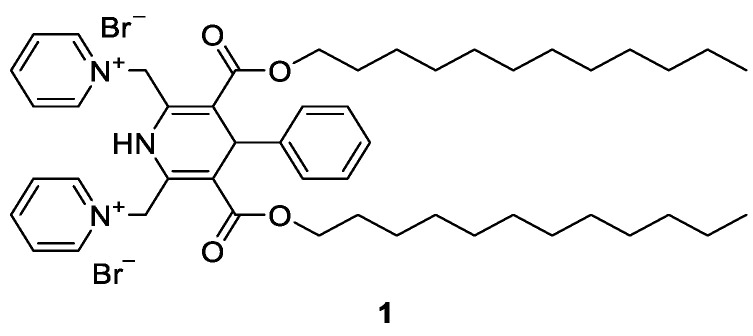
Structure of 1,4-DHP amphiphile—1,1′-{[3,5-bis(dodecyloxycarbonyl)-4-phenyl-1,4-dihydropyridine-2,6-diyl]bis(methylene)}bis(pyridin-1-ium) dibromide (**1**).

Previous studies of our research group have demonstrated that 1,4-dihydropyridines (1,4-DHPs) containing pyridinium salts as substituents demonstrated self-assembling properties and formed liposomes. Some of these class members showed DNA delivery potential. Several representatives (for example, comp.**1**; Figure 1) appeared to be more active for DNA delivery than commercially available cationic lipid DOTAP (*N*-(1-(2,3-dioleoyloxy)propyl)-*N*,*N*,*N*-trimethyl ammonium methylsulfate) and polymer PEI 25 (polyethyleneimine of 25 kDa) and have potential for use as transmembrane delivery systems [16,17]. The mentioned amphiphiles contain 1,4-DHP group as an active linker and, as stated by Triggle [18], are an intrinsic structural part of many pharmacologically active compounds and drugs.

The first metabolic step of 1,4-DHPs is their oxidation to the pyridines. Therefore, it was rational to investigate the properties of oxidized forms of the previously studied 1,4-DHP lipids. Synthetic polyfunctional lipids on a base of pyridines have been designed and studied for the evaluation of structure–activity relationships [19]. The latest data have confirmed that cationic moieties remoted from the linker change the delivery activity of cationic 1,4-DHPs [20]. The magnetoliposomes comprised from liposomes filled with magnetic iron oxide nanoparticles [21] and magnetic nanoparticles modified with pyridinium moieties containing 1,4-DHP demonstrated bactericidal and immunomodulatory properties [22]. Recently, the antiproliferative activity of amphiphilic 1,4-DHP has been described against cancer cell lines HT-1080 and MH-22A, along with low cytotoxicity towards non-cancerous NIH3T3 cells [23]. Some fluorescent fragment containing 1,4-DHP amphiphiles—a derivative with 4-(2-(naphthalen-2-yl)vinyl)pyridinium moieties [24] and 4-(anthracen-9-yl)pyridinium ones [25]—were obtained and characterized. The photoluminescence quantum yield values for the EtOH solution of these compounds were 1.0 and 10.8%, respectively. In this respect, synthesized synthetic lipids have the potential to be used in the development of prospective theragnostic delivery systems.

As a part of our current research program towards the development of novel synthetic delivery systems, we have continued studies to establish structure–activity relationships. In this work we have evaluated the influence of *N*-benzyl substituent at the 1,4-dihydropyridine ring on physicochemical and self-assembling properties of the parent compound. We have performed characterization of monolayers composed of the tested amphiphiles, estimated self-assembling properties, and described the nanoparticles and lipoplexes formed by the amphiphiles. All the data acquired for the novel *N*-benzyl 1,4-DHPs were compared to the *N*-unsubstituted 1,4-DHP **1** as a control. The results may help to clarify the further conception of the structure–activity and liposome parameter relationships of 1,4-DHP amphiphiles as prospective delivery systems.

## 2. Materials and Methods

### 2.1. General

All reagents were purchased from Acros Organics (Geel, Belgium), Alfa Aesar (Lancashire, UK), or Sigma-Aldrich/Merck KGaA (Darmstadt, Germany), and were used without further purification. Tin layer chromatography (TLC) was performed on silica gel 60 F_254_ aluminum sheets 20 cm × 20 cm (Merck KGaA, Darmstadt, Germany) and visualized under UV (254 nm) light and was used to monitor reaction progress. Silica gel of particle size 35–70 µm (Merck KGaA, Darmstadt, Germany) was used for column chromatography for purifications of the compounds. Melting points (mp) were determined on an OptiMelt digital melting point apparatus (Stanford Research Systems, Sunnyvale, CA, USA) and were uncorrected. Proton Nuclear Magnetic Resonance (^1^H NMR) spectra were recorded with a Bruker Avance Neo (400 MHz) spectrometer and Carbon Nuclear Magnetic Resonance (^13^C NMR) spectra were recorded with a Bruker Avance Neo (101 MHz) spectrometer (Bruker Biospin Gmbh, Rheinstetten, Germany). The obtained data were used for identification of compound structures. Chemical shifts of the hydrogen and carbon atoms are presented in parts per million (ppm) and referred to as the residual signals of the non-deuterated CDCl_3_ (δ: 7.26) or partially deuterated DMSO-*d*_6_ (δ: 2.50) solvents for the ^1^H NMR spectra and CDCl_3_ (δ: 77.16) or DMSO-*d*_6_ (δ: 39.5) solvents for ^13^C NMR, respectively. Coupling constants *J* were reported in hertz (Hz). High resolution mass spectra (HRMS) were determined on an Acquity Ultra High Performance Liquid Chromatography (UPLC) H-Class system (Waters, Milford, MA, USA) connected to a Waters Synapt GII Q-ToF operating in the ESI positive ion mode on a Waters Acquity UPLC^®^ BEH C18 column (1.7 µm, 2.1 mm × 50 mm, using gradient elution with acetonitrile (0.1% formic acid) in water (0.1% formic acid). High Resolution Mass Spectrometry (HRMS) data were used to determine the exact molecular masses of compounds. Elemental analyses were determined on an Elemental Combustion System ECS 4010 (Costech International S.p.A., Milano, Italy). Infrared spectra (IS) were recorded with a Prestige-21 FTIR spectrometer (Shimadzu, Kyoto, Japan) and were used for structure confirmation. Flash column chromatography was accomplished using flow chromatography (Armen Instrument—Spot Flash System Interchim, Montluçon, France). The characterization of monolayers was performed by Langmuir trough (Medium trough, KSV NIMA Instruments, Espoo, Finland; A_total_ = 243 cm^−2^). The Dynamic Light Scattering (DLS) measurements of the nanoparticles in the aqueous solution were carried out on a Zetasizer Nano ZSP (Malvern Panalytical Ltd., Malvern, UK) instrument with Malvern Instruments Ltd. Software 8.01.4906.

### 2.2. Synthesis of Compounds

#### 2.2.1. Didodecyl 1-Benzyl-2,6-dimethyl-4-phenyl-1,4-dihydropyridine-3,5-dicarboxylate (**2**)

A solution of dodecyl acetoacetate (10.3 g, 0.038), phenylmethanamine hydrochloride (2.73 g, 0.019 mol) and benzaldehyde (2.00 g, 0.19 mol, 1.90 mL) in pyridine (20 mL) was refluxed for 30 h. The reaction mixture was poured into an ice/water mixture (~100 mL), extracted with CH_2_Cl_2_ (3 × 30 mL), and the organic phase was washed with 1N HCl (3 × 30 mL). The solvent was evaporated in vacuo, after which the residue was crystallized from MeOH and dried in vacuo. The desired product was obtained as a pale-yellow solid (6.17 g, 46%), mp 102–104 °C. *R_f_* = 0.23 (EtOAc:PE, 1:10).

^1^H NMR (400 MHz, CDCl_3_) δ (ppm): 7.24–7.12 (m, 8H), 6.96–6.92 (m, 2H), 5.23 (s, 1H), 4.85 (s, 2H), 4.09 (t, *J* = 6.6 Hz, 4H), 2.43 (s, 6H), 1.67–1.58 (m, 4H), 1.31–1.23 (m, 36H), 0.88 (t, *J* = 6.9 Hz, 6H). ^13^C NMR (101 MHz, CDCl_3_) δ (ppm): 168.36, 148.89, 146.58, 137.98, 128.93, 128.08, 127.40, 126.19, 126.08, 107.09, 64.34, 49.69, 38.29, 32.06, 29.82, 29.79, 29.77, 29.72, 29.50, 29.45, 28.87, 26.24, 22.83, 16.83, 14.25. IR ν_max_ (film) 2917, 2849, 1695, 1671, 1629, 1570 cm^−1^. HRMS TOF ES^+^ of [C_46_H_69_NO_4_-2H+H]^+^ (*m*/*z*) 698.5155; calcd: 698.5148. Anal. calcd. for C_46_H_69_NO_4_: C, 78.92; H, 9.94; N, 2.00; found: C, 78.90; H, 10.14; N, 2.01.

#### 2.2.2. Didodecyl 1-Benzyl-2,6-bis(bromomethyl)-4-phenyl-1,4-dihydropyridine-3,5-dicarboxylate (**3**)

To a solution of didodecyl 1-benzyl-2,6-dimethyl-4-phenyl-1,4-dihydropyridine-3,5-dicarboxylate (**2**) (1.0 g, 140 mmol) in methanol (250 mL), a solution of NBS (0.51 g, 285 mmol) in methanol (50 mL) was added dropwise. The reaction mixture was stirred at rt for 2 h, and the completion of the reaction was monitored by TLC. The reaction mixture was cooled to 4 °C, precipitate was filtered off and washed with water, and the crude product was purified by flash column chromatography with gradient elution: EtOAc:hexane 0:100 to 15:85%, to give the desired product **3** as a yellow solid (0.88 g, 74%), mp 63–65 °C. *R_f_* = 0.47 (EtOAc:PE, 1:10).

^1^H NMR (400 MHz, CDCl_3_) δ (ppm): 7.25–7.21 (m, 3H), 7.19–7.13 (m, 3H), 7.07 (t, *J* = 7.5 Hz, 2H), 6.66 (d, *J* = 7.5 Hz, 2H), 5.38 (s, 1H), 5.12 (s, 2H), 4.95 (br. s, 2H), 4.73 (br. s, 2H), 4.24–4.14 (m, 4H), 1.71–1.63 (m, 4H), 1.33–1.23 (m, 36H), 0.88 (t, *J* = 6.8 Hz, 6H). ^13^C NMR (101 MHz, CDCl_3_) δ (ppm): 166.72, 147.46, 144.21, 137.06, 129.14, 128.37, 127.81, 127.18, 126.81, 126.06, 109.42, 65.32, 48.49, 37.59, 32.07, 29.82, 29.79, 29.75, 29.71, 29.51, 29.42, 28.76, 26.20, 25.12, 22.84, 14.27. IR ν_max_ (film) 3064, 2952, 2925, 2854, 1694, 1624, 1576 cm^−1^. HRMS TOF ES^+^ of [C_46_H_67_NO_4_Br_2_+H]^+^ (*m*/*z*) 856.3491; calcd: 856.3515. Anal. calcd. for C_46_H_67_Br_2_NO_4_: C, 64.41; H, 7.87; N, 1.63; found: C, 64.03; H, 7.86; N, 1.67.

#### 2.2.3. General Procedure for Synthesis of 1,1′-((1-Benzyl-3,5-bis(dodecyloxycarbonyl)-4-phenyl-1,4-dihydropyridine-2,6-diyl)bis(methylene))bis(pyridin-1-ium) Dibromides **5**

To a stirred solution of didodecyl 1-benzyl-2,6-bis(bromomethyl)-4-phenyl-1,4-dihydropyridine-3,5-dicarboxylate (**3**, 0.17 mmol) in acetone (10 mL) at r.t., the corresponding pyridine derivative (0.35 mmol) was added, and the resulting mixture was stirred at r.t. for 24 h. The reaction was monitored by TLC. The reaction mixture was cooled to 4 °C, and the precipitates were filtered off, washed with cold acetone, and dried in vacuo to give the desired dibromides **5**. 

##### 1,1′-((1-Benzyl-3,5-bis((dodecyloxy)carbonyl)-4-phenyl-1,4-dihydropyridine-2,6-diyl)bis(methylene))bis(pyridin-1-ium) Dibromide **5a**

Pale yellow solid, yield 35%, mp 147 °C (decomp.). *R_f_* = 0.21 (sat. KNO_3_:H_2_O:Acetone, 1:4:80).

^1^H NMR (400 MHz, DMSO-*d*_6_) δ (ppm): 8.88 (d, *J* = 6.2 Hz, 4H), 8.55 (t, *J* = 7.7 Hz, 2H), 8.07 (dd, *J* = 7.7, 6.2, Hz, 4H), 7.29–7.19 (m, 3H), 7.19–7.13 (m, 3H), 7.02 (t, *J* = 7.6 Hz, 2H), 6.75 (d, *J* = 7.2 Hz, 2H), 6.39 (AB-system, *J* = 16.3 Hz, 2H), 5.79 (AB-system, *J* = 16.3 Hz, 2H), 5.31 (s, 1H), 4.81 (s, 2H), 4.17–4.07 (m, 4H), 1.59–1.50 (m, 4H), 1.26–1.18 (m, 36H), 0.85 (t, *J* = 6.6 Hz, 6H). ^13^C NMR (101 MHz, DMSO-*d*_6_) δ (ppm): 165.67, 146.24, 144.12, 142.74, 141.15, 135.80, 128.58, 128.31, 128.25, 127.87, 126.99, 126.97, 126.92, 115.45, 65.31, 55.33, 48.39, 40.15, 39.94, 39.73, 39.52, 39.31, 39.10, 38.89, 38.23, 31.31, 29.08, 29.02, 28.99, 28.73, 28.69, 27.92, 25.57, 22.10, 13.95. IR ν_max_ (film) 2952, 2922, 2852, 1719, 1699, 1631, 1594 cm^−1^. HRMS TOF ES^+^ of [C_56_H_77_N_3_O_4_-2H+H]^+^ (*m*/*z*) 854.5842; calcd: 854.5836. Anal. calcd. for C_56_H_77_Br_2_N_3_O_4_: C, 66.20; H, 7.64; N, 4.14; found: C, 66.15; H, 7.58; N, 4.25.

##### 1,1′-((1-Benzyl-3,5-bis((dodecyloxy)carbonyl)-4-phenyl-1,4-dihydropyridine-2,6-diyl)bis(methylene))bis(4-methylpyridin-1-ium) **5b**

Pale yellow solid, yield 50%, mp 179 °C (decomp.). *R_f_* = 0.28 (sat. KNO_3_:H_2_O:Acetone, 1:4:80).

^1^H NMR (400 MHz, DMSO-*d*_6_) δ (ppm): 8.72 (d, *J* = 6.7 Hz, 4H), 7.93 (d, *J* = 6.7 Hz, 4H), 7.29–7.13 (m, 6H), 7.02 (t, *J* = 7.7 Hz, 2H), 6.72 (d, *J* = 6.9 Hz, 2H), 6.34 (AB-system, *J* = 16.2 Hz, 2H), 5.64 (AB-system, *J* = 16.3 Hz, 2H), 5.29 (s, 1H), 4.69 (s, 2H), 4.20–4.06 (m, 4H), 2.59 (s, 6H), 1.60–1.51 (m, 4H), 1.28–1.16 (m, 36H), 0.86 (t, *J* = 6.9 Hz, 6H). ^13^C NMR (101 MHz, DMSO-*d*_6_) δ (ppm): 165.72, 159.72, 143.11, 142.82, 141.19, 135.82, 128.53, 128.20, 127.78, 126.99, 126.95, 126.90, 115.42, 65.27, 54.48, 48.16, 38.23, 31.31, 29.08, 29.02, 28.99, 28.73, 28.70, 27.94, 25.60, 22.10, 21.29, 13.94. IR ν_max_ (film) 3030, 2954, 2922, 2854, 1695, 1640, 1594 cm^−1^. HRMS TOF ES^+^ of [C_58_H_81_N_3_O_4_-2H+H]^+^ (*m*/*z*) 882.6138; calcd: 882.6149. Anal. calcd. for C_58_H_81_Br_2_N_3_O_4_: C, 66.72; H, 7.82; N, 4.02; found: C, 66.43; H, 7.69; N, 4.21.

##### 1,1′-((1-Benzyl-3,5-bis((dodecyloxy)carbonyl)-4-phenyl-1,4-dihydropyridine-2,6-diyl)bis(methylene))bis(4-(dimethylamino)pyridin-1-ium) **5c**

Pale yellow solid, yield 75%, mp 158 °C (decomp.). *R_f_* = 0.20 (sat. KNO_3_:H_2_O:Acetone, 1:4:80).

^1^H NMR (400 MHz, DMSO-*d*_6_) δ (ppm): 8.11 (d, *J* = 7.8 Hz, 4H), 7.28–7.13 (m, 4H), 7.12–7.09 (m, 2H), 7.00 (t, *J* = 7.8 Hz, 2H), 6.91 (d, *J* = 7.8 Hz, 4H), 6.68 (d, *J* = 7.2 Hz, 2H), 5.98 (AB-system, *J* = 16.0 Hz, 2H), 5.27 (AB-system, *J* = 16.0 Hz, 2H) overlap, 5.26 (s, 1H) overlap, 4.68 (s, 2H), 4.20–4.08 (m, 4H), 3.18 (s, 12H), 1.61–1.52 (m, 4H), 1.28–1.16 (m, 36H), 0.87 (t, *J* = 6.8 Hz 6H). ^13^C NMR (101 MHz, DMSO-*d*_6_) δ (ppm): 165.93, 155.83, 143.07, 142.53, 141.22, 135.99, 128.45, 128.13, 127.65, 126.89, 126.83, 126.75, 114.40, 107.85, 65.15, 51.36, 47.94, 39.85, 38.07, 31.30, 29.07, 29.01, 28.99, 28.72, 28.70, 27.97, 25.62, 22.10, 13.94. IR ν_max_ (film) 3029, 2955, 2924, 2853, 1694, 1651, 1574 cm^−1^. HRMS TOF ES^+^ of [C_60_H_87_N_5_O_4_-2H+H]^+^ (*m*/*z*) 940.6695 calcd: 940.6680. Anal. calcd. for C_60_H_87_Br_2_N_7_O_4_: C, 65.38; H, 7.96; N, 6.35; found: C, 65.02; H, 7.67; N, 6.50.

##### 1,1′-((1-Benzyl-3,5-bis((dodecyloxy)carbonyl)-4-phenyl-1,4-dihydropyridine-2,6-diyl)bis(methylene))bis(4-phenylpyridin-1-ium) **5d**

Pale yellow solid, yield 53%, mp 180 °C (decomp.). *R_f_* = 0.60 (sat. KNO_3_:H_2_O:Acetone, 1:4:80).

^1^H NMR (400 MHz, DMSO-*d*_6_) δ (ppm): 8.98 (d, *J* = 6.8 Hz, 4H), 8.46 (d, *J* = 6.8 Hz, 4H), 7.92–7.86 (m, 4H), 7.66–7.60 (m, 2H), 7.52–7.46 (m, 4H), 7.31–7.17 (m, 6H), 7.04 (t, *J* = 7.7 Hz, 2H), 6.76 (d, *J* = 7.2 Hz, 2H), 6.46 (AB-system, *J* = 16.3 Hz, 2H), 5.69 (AB-system, *J* = 16.3 Hz, 2H), 5.32 (s, 1H), 4.73 (s, 2H), 4.24–4.11 (m, 4H), 1.60–1.52 (m, 4H), 1.27–1.12 (m, 36H), 0.85 (t, *J* = 6.8 Hz, 6H). ^13^C NMR (101 MHz, DMSO-*d*_6_) δ (ppm): 165.84, 155.33, 144.29, 142.85, 140.59, 135.92, 132.89, 132.34, 129.52, 128.57, 128.17, 128.09, 127.78, 127.05, 127.01, 126.88, 124.51, 115.89, 65.39, 54.51, 48.13, 38.35, 31.30, 29.05, 29.00, 28.74, 28.71, 27.97, 25.68, 22.10, 13.95. IR ν_max_ (film) 3032, 2952, 2924, 2854, 1694, 1639, 1597 cm^−1^. HRMS TOF ES^+^ of [C_68_H_85_N_3_O_4_-2H+H]^+^ (*m*/*z*) 1006.6453; calcd: 1006.6462. Anal. calcd. for C_68_H_85_Br_2_N_3_O_4_: C, 69.91; H, 7.33; N, 3.60; found: C, 69.65; H, 7.16; N, 3.87.

##### 1,1′-((1-Benzyl-3,5-bis((dodecyloxy)carbonyl)-4-phenyl-1,4-dihydropyridine-2,6-diyl)bis(methylene))bis(4-propylpyridin-1-ium) **5e**

Pale yellow solid, yield 41%, mp 176 °C (decomp.). *R_f_* = 0.50 (sat. KNO_3_:H_2_O:Acetone, 1:4:80).

^1^H NMR (400 MHz, DMSO-*d*_6_) δ (ppm): (d, *J* = 6.5 Hz, 4H), 7.97 (d, *J* = 6.5 Hz, 4H), 7.30–7.15 (m, 6H), 7.00 (t, *J* = 7.7 Hz, 2H), 6.70 (d, *J* = 6.8 Hz, 2H), 6.30 (AB-system, *J* = 16.3 Hz, 2H), 5.66 (AB-system, *J* = 16.3 Hz, 2H), 5.29 (s, 1H), 4.76 (s, 2H), 4.18–4.06 (m, 4H), 2.87–2.77 (m, 4H), 1.71–1.60 (m, 4H), 1.57–1.49 (m, 4H), 1.25–1.18 (m, 36H), 0.93 (t, *J* = 7.3 Hz, 6H), 0.85 (t, *J* = 6.8 Hz, 6H). ^13^C NMR (101 MHz, DMSO-*d*_6_) δ (ppm): 165.68, 163.24, 143.32, 142.90, 140.80, 135.93, 128.51, 128.21, 127.77, 127.74, 127.03, 126.93, 126.77, 115.49, 65.27, 54.65, 48.27, 38.23, 36.42, 31.31, 29.08, 29.02, 28.99, 28.73, 28.70, 27.92, 25.59, 22.74, 22.10, 13.94, 13.45. IR ν_max_ (film) 3029, 2956, 2924, 2853, 1696, 1638, 1596 cm^−1^. HRMS TOF ES^+^ of [C_62_H_89_N_3_O_4_-2H+H]^+^ (*m*/*z*) 938.6781; calcd: 938.6775. Anal. calcd. for C_62_H_89_Br_2_N_3_O_4_: C, 67.68; H, 8.15; N, 3.82; found: C, 67.34; H, 7.96; N, 3.98.

### 2.3. Characterization of Monolayers Formed by 1,4-DHP Amphiphiles ***5*** or Surface Pressure–Area (π–A) Isotherms

The surface pressure–molecular area (π–A) compression isotherms were measured using a computer-controlled Langmuir trough (Medium trough, KSV NIMA Instruments, Finland; A_total_ = 243 cm^−2^) made of Teflon and equipped with two compression barriers according to the previously described method [21]. Prior to the measurements, the trough and barriers were thoroughly rinsed with ethanol and Milli-Q water. Briefly, the surface pressure of the monolayer was monitored with a Wilhelmy plate made of platinum, which was cleaned by being flushed with ethanol and Milli-Q water and burned by a Bunsen burner. Samples for the studies were prepared by making stock solutions of the compounds in CHCl_3_ at a concentration of 1 mg/mL and 0.125 mg/mL for compound **5a**.

Cleanliness of the aqueous surface was ensured by sweeping the barriers across the surface, and the aqueous surface was considered clean when π ≤ 0.2 mN/m. Monolayers were formed by carefully spreading an appropriate volume of the lipid solution in chloroform (20 μL, for compound **5e**—10 μL) dropwise on the deionized water surface at 25 ± 1 °C using a Hamilton micro-syringe. The carrying solvent (CHCl_3_) was allowed to evaporate for 5 min before compressions began. The monolayers were compressed at a constant rate of 10 mm/min. Measurements were made at 25 ± 1 °C and repeated at least three times to ensure the reproducibility of the results. The experimentally detected standard deviations of the molecular area and surface pressure did not exceed 5%.

### 2.4. Self-Assembling Properties of Compounds ***5*** Using Dynamic Light Scattering Measurements

Self-assembling properties of the tested compounds were estimated according to a published procedure with minor modifications [17,25]. Briefly, samples for the DLS studies were prepared by making stock solutions of compounds **5** in EtOH at a concentration of 0.4 mM. Stock solutions of the compounds (2.5 mL, 0.4 mM in EtOH 96%) were injected into 7.5 mL of deionized water with maximum stirring (IKA Vortex 2 (IKA, Staufen, Germany)) to give samples a final compound concentration of 0.1 mM.

The DLS measurements of the nanoparticles in the aqueous solution were carried out on a Zetasizer Nano ZSP (Malvern Panalytical Ltd., Malvern, UK) instrument with Malvern Instruments Ltd. Software 8.01.4906, using the following specifications—medium: water; refractive index: 1.33; viscosity: 0.8872 cP; temperature: 25 °C; dielectric constant: 78.5; nanoparticles: liposomes; refractive index of materials: 1.60; detection angle: 173°; wavelength: 633 nm. Data were analyzed using the multimodal number distribution software that was provided with the instrument. The measurements were performed in triplicate to ensure their reproducibility.

### 2.5. Determination of Critical Aggregation Concentrations

Critical aggregation concentrations of the tested compounds were estimated according to a published procedure with minor modifications [17]. The critical aggregation concentrations (CAC) of 1,4-DHP amphiphile **1** and *N*-benzyl 1,4-DHP pyridinium dibromides **5** were detected using a Zetasizer Nano S90 (Malvern Panalytical Ltd., Malvern, UK) instrument with Malvern Instruments Ltd. The software procedure was described by Topel et al. and modified by our group [17,26]. Briefly, stock solutions of the compounds (0.3 mL, 0.94 mM in EtOH 96%) were injected into 0.7 mL of phosphate-buffered saline (PBS) (pH = 7.4) with maximum stirring (IKA Vortex 2 (IKA, Staufen, Germany)) to give samples with a final concentration of the compound of 0.282 mM. All of the subsequent samples were prepared starting from this concentrated solution, which was subjected to a serial two-fold dilution each time with PBS (pH = 7.4). The intensity values of scattered light (kcps) as a function of concentration of amphiphiles were analyzed. The scattering intensities detected for amphiphile concentrations below CAC have an approximately constant value that corresponds to water. The intensity starts to show a linear increase with concentration at the CAC, since the number of nanoparticles increases in the solution. The intersection of the best-fit lines drawn through the data points is the preliminary CAC value of the compounds.

### 2.6. Formation of Lipoplexes

The lipoplexes were prepared by placing 9 µL of 0.04 µg/uL mRNA solution into 2 mL sterile Eppendorf tube, followed by the addition of 6 µL of lipid solution in ethanol with three different concentrations—0.92 mM, 1.84 mM, and 4.6 mM—to obtain N/P charge ratios of 1, 2, and 5, following immediate mixing by working the pipette up-and-down several times. The N/P charge ratio is nitrogen/phosphate ratio, which means that cationic 1,4-DHP amphiphiles were mixed with mRNA at different charge proportions. The obtained solutions were incubated for 15 min and 345 µL of Minimum Essential Medium Eagle (MEM) were added to each formulation. Solutions were mixed by vortexing and were incubated for 30 min before DLS measurements.

### 2.7. Statistical Analysis

Results were expressed as mean standard deviations (SDs). All of the experiments were performed in triplicate.

## 3. Results and Discussion

### 3.1. Synthesis of N-Benzyl-1,4-dihydropyridines

**Scheme 1:**
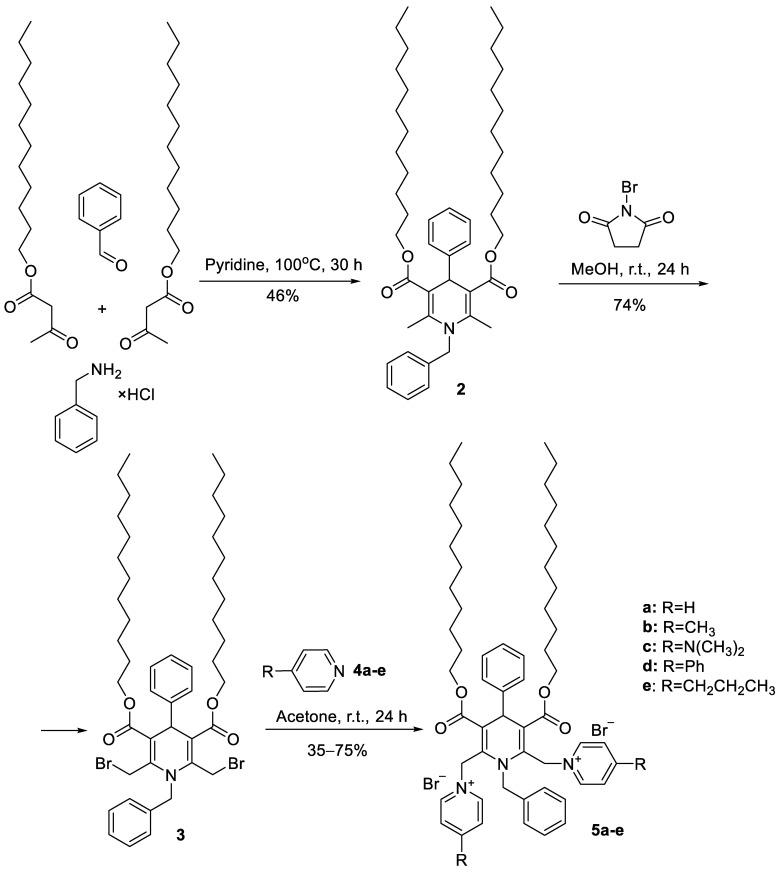
Synthesis of 1,1′-((1-benzyl-3,5-bis(dodecyloxycarbonyl)-4-phenyl-1,4-dihydropyridine-2,6-diyl)bis(methylene))bis(pyridin-1-ium) dibromides **5**.

The synthetic strategy for synthesis of the target products is depicted in Scheme 1.

The variations in the structure of original *N*-benzyl 1,4-DHP amphiphiles **5** were performed in two directions: (1) by introducing the benzyl substituent at N atom of 1,4-DHP cycle in order to evaluate the influence of *N*-substituent (comp. **1** versus comp. **5a**); (2) by variation of pyridinium substituents in order to study the influence of cationic head groups on the properties of amphiphiles (comp. **5a** versus comp. **5b–e**).

Firstly, a parent *N*-benzyl 1,4-dihydropyridine **2** was synthesised by the condensation of benzaldehyde, with two equivalents of dodecyl acetoacetate using phenylmethanamine hydrochloride as a nitrogen source instead of ammonia and pyridine as a solvent, under reflux for 30 h. Bromination of the 2,6-methyl groups of the *N*-benzyl 1,4-DHP **2** was performed by *N*-bromosuccinimide (NBS), similarly to a previously described procedure [27]. A solution of NBS in methanol was added dropwise to a solution of 2,6-dimethyl-1,4-DHP **2** in methanol, the reaction mixture was stirred at r. t. for 24 h, and the completion of the reaction was monitored by TLC. The desired *N*-benzyl substituted 2,6-bis(bromomethyl)-1,4-DHP **3** was obtained with a yield of 74%. The last step—bromine nucleophilic substitution with pyridine derivatives—was performed in acetone by stirring the reaction mixture at the room temperature for 24 h. This step gave the target *N*-benzyl 1,4-DHP amphiphiles **5** with yields of 35–75% depending on the pyridinium substituent structure.

The structures of all compounds were established and confirmed on the base of the ^1^H NMR (Figures S1, S4, S7, S10, S13, S16 and S19, Supplementary Materials), ^13^C NMR (Figures S2, S5, S8, S11, S14, S17 and S20, Supplementary Materials), IR, HRMS (Figures S3, S6, S9, S12, S15, S18 and S21, Supplementary Materials), and elemental analysis data. ^1^H NMR spectra of all compounds showed characteristic signals for 1,4-DHP 4-H protons as singlets at a range of 5.23–5.38 ppm and *N*-benzyl-CH_2_- signals as singlets in the range of 4.68–5.12 ppm. Additionally, characteristic AB-system signals from the 2,6-methylene protons were observed for all *N*-benzyl 1,4-DHP pyridinium dibromides **5** in the ^1^H NMR spectra in the range of 5.27–5.79 and 5.98–6.39 ppm, while the same protons for *N*-benzyl substituted 2,6-bis(bromomethyl)-1,4-DHP **3** were observed as two broad singlets at 4.75 and 4.95 ppm. Characteristic signals of 4-H proton and AB-system signals from the 2,6-methylene protons of 1,4-DHP derivatives are in agreement with the data for the previously published structurally related compounds. Thus, amphiphilic 1,4-DHP-anthracene hybrid signals of 1,4-DHP 4-H were observed at 5.15 ppm, and the 2,6-methylene group AB-system signals were observed at 5.89 and 6.39 ppm [25]. The ^1^H NMR spectra of amphiphilic 1,4-DHP derivative having 4-(naphthalen-2-yl)-substituent at position 4 and (*E*)-4-(2-(naphthalen-2-yl)vinyl)pyridinium substituents at positions 2 and 6 of 1,4-DHP cycle demonstrated signals at 6.50 ppm for 4-H proton and 4.67 and 5.44 ppm for 2,6-methylene group protons [24].

In the IR spectra, 1,4-DHP derivatives **2**, **3**, and **5** demonstrated characteristic absorption bands in the region of 3060–2850 cm^−1^ characteristic for long alkyl chains and absorptions in 1720–1600 cm^−1^ of its C=O and C=C groups.

HRMS data of *N*-benzyl-1,4-DHP **2** and all cationic moieties containing 1,4-DHP amphiphiles **5a–e** showed mass signals which correspond to oxidized forms of the compounds, while *N*-benzyl substituted 2,6-bis(bromomethyl)-1,4-DHP **3** mass signal confirms 1,4-DHP form.

The purities of the studied compounds **5a–e** were at least 97% according to high-performance liquid chromatography (HPLC) analysis.

### 3.2. Characterization of Monolayers

The properties of the monolayers composed of *N*-unsubstituted 1,4-DHP amphiphile **1** and *N*-benzyl-1,4-DHP derivatives **5** and their polar head areas were determined from π–A isotherms obtained using the Langmuir–Blodgett trough (Figure S22, Supplementary Materials) and were calculated according to the previously published procedures [21]. To analyze the physical state of the monolayers, the values of compressibility modulus (C_s_^−1^) were calculated from the isotherm data (Figure S23, Supplementary Materials). The surface pressure (P) was defined as the surface pressure at C_s_^−1^_max_. The mean molecular area (MMA) of the compound of interest could be extracted from the π−A isotherm in the liquid-condensed phase. The obtained values of the mentioned parameters are listed in Table 1.

As observed from the π−A isotherms, all the studied compounds were able to form stable monolayers in an aqueous medium. The mean molecular area of the *N*-unsubstituted 1,4-DHP **1** was 96 Å^2^ (Table 1, entry 1). This was in agreement with the previously published data, where the MMA value for 1,4-DHP **1** was indicated as 89 Å^2^ [25] or 83 Å^2^ [21]. After the introduction of *N*-benzyl substituent to 1,4-DHP molecules, the values of mean molecular area increased from 132 to 139 Å^2^ (Table 1, entries 2–5) for compounds **5a**–**d**, which confirms that, in the mentioned cases, the substituents at pyridinium moieties do not make significant contributions to change the mean molecular area value. The MMA value for compound **5e** enlarged twice, reaching the value of 191 Å^2^ (Table 1, entry 6) and demonstrating the influence of the *N*-benzyl substituent and substituents at pyridinium moieties. There are three clearly distinguished zones in the Langmuir isotherm of compound **5e** which are attributed to the structural characteristics—linear propyl substituent at position 4 of pyridinium moieties, which decreases the hydrophilicity of the polar head group. A similar isotherm with five zones was registered for dendrimers and showed the influence of the hydrophilic/hydrophobic moiety ratio on surface phase behavior [28]. According to the C_s_^−1^_max_ values and the literature data, all of the formed monolayers collapsed in the liquid-condensed phase [29].

There are three clearly distinguished zones in the Langmuir isotherm of compound **5e** which are related to the structural characteristics. The linear propyl substituent at position 4 of pyridinium moieties decreases the hydrophilicity of the polar head group.

### 3.3. Self-Assembling Properties

Self-assembling properties are a characteristic feature of synthetic lipid-like compounds, including 1,4-DHPs, which contain cationic moieties. The average hydrodynamic diameters (Z_av_), polydispersity index (PDI), zeta-potential (Z_pot_), critical aggregation concentrations (CAC), and stability of nanoparticles formed by *N*-benzyl-1,4-DHP amphiphiles **5** in aqueous medium were determined by the DLS method. Nanoparticles were prepared by the ethanol injection method, which involved the dissolution of the lipid-like compound into organic solvent followed by dispersion of the lipid solution into water. This method is rapid and easily scalable [30]. Nanoparticles were prepared from cationic moieties containing *N*-benzyl-1,4-DHP amphiphiles **5**. Parent 1,4-DHP derivatives **2** and **3**, which were used for the preparation of derivatives **5**, do not form nanoparticles by themselves. The same relationships were observed previously for N-unsubstituted ones [17].

The data concerning zeta-potential (Z_pot_) and CAC are summarized in Table 2.

The obtained data indicated that the surface charges of nanoparticles formed by tested amphiphiles were strongly positive. Surface charge of lipid nanoparticles is usually determined by the lipid head groups. The surface potential of nanoparticles determines the strength of intra-particle interactions, the adsorption of counter ions, and particle stability. Zeta-potential values over 30 mV usually indicate that the formed nanoparticle solutions are relatively stable [31,32]. The Z_pot_ value of compound **1**—around 75 mV (Table 2, entry 1)—is in agreement with our previous measurements when the zeta-potential value of compound **1** was indicated as 82 mV [17] or 90 mV [19]. Z_pot_ values of *N*-benzyl 1,4-DHP derivatives **5b** and **5e** are relatively close—89 and 91 mV, respectively. Z_pot_ values of other *N*-benzyl 1,4-DHP derivatives **5a**, **5c**, and **5d** are lower, in the range 40, 57, and 36 mV, respectively (Table 2, entry 1).

For self-assembling compounds, the concentration above which vesicles, micelles, and other nanoparticles are formed is described as the critical micelle concentration (CMC), which is an important parameter for the characterization of the compounds of this type [33]. The point where amphiphiles assemble in larger spherical aggregates is CMC, while the concentration of premicellar aggregates formation is critical aggregation concentration (CAC). Compared to the monomeric analogues, gemini surfactants show more diverse aggregate morphologies [34]. Therefore, the term CAC was used for the characterization of a critical concentration of 1,4-DHP amphiphiles **1** and **5**. According to Felice et al. [35] for the conventional amphiphiles, CMC is usually in the range of 10^−2^–10^−4^ M. Alternatively, for phospholipids, CMC is four to five times smaller, which indicates that these materials have rather low solubility in water. Because of this, phospholipids have higher stability after administration when compared to micelles. CAC values of 1,4-DHP amphiphiles **1** and **5** were in the range of 0.01–2.65 µM (Table 2, entry 2). The CAC value of 1,4-DHP amphiphile **1** was 1.06 µM, while the previously determined CAC values were 30 µM [17] or 17 µM [19]. This variance could be explained by the different sample preparation methodology. Previously, nanoparticles were prepared by thin film hydration method in aqueous media, while this time the ethanol injection method was used. With this method, the nanoparticle media contains a residual amount of ethanol additive. CAC values of *N*-benzyl 1,4-DHPs **5b** and **5d** are in the same range—1.16 µM and 0.95 µM. CAC values of other *N*-benzyl 1,4-DHPs are 2.65 µM for comp. **5d**, 0.46 µM for comp. **5a**, and 0.01 µM for comp. **5c**.

#### 3.3.1. Characterization of Size of Liposomes

**Figure 2:**
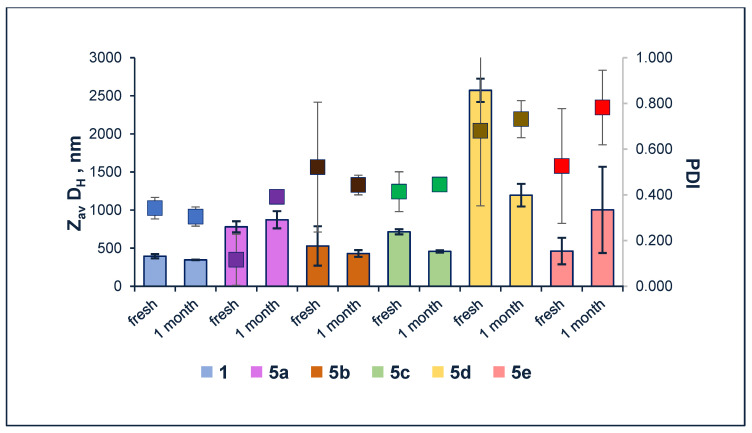
Values of Z-average (Z_av_D_H_) diameter and polydispersity index (PDI) of nanoparticles formed by 1,4-DHP amphiphiles **1** and **5** at the 0.1 mM concentration obtained by DLS measurements for the freshly prepared samples and samples after one month storage at 4 °C. Error bars smaller than data point size are not visible.

The average hydrodynamic diameters (Z_av_D_H_), polydispersity index (PDI), and stability of nanoparticles comprised of amphiphilic 1,4-DHP **1** and **5** were determined by the DLS method. The results for the freshly prepared samples and samples after storage at 4 °C for a month at concentration 0.1 mM are summarized in Figure 2 (data from Table S1, Supplementary Materials).

The average diameter of nanoparticles formed by *N*-benzyl 1,4-DHP derivatives **5** in an aqueous medium ranged from 395 to 2570 nm for the freshly prepared samples and from 346 to 1195 nm after one month of storage (Figure 2). The structure of the compounds, including the substituent of pyridinium moieties, influenced the values of nanoparticles’ average diameter. Thus, in the case of bulky phenyl substituent for compound **5d**, the average diameter of nanoparticles was 2570 nm, and the PDI value of 0.680 confirmed the heterogeneity of the sample. After one month of storage the diameter of particles decreased twice (to 1195 nm), but the PDI value increased to 0.731. Nanoparticles formed by *N*-unsubstituted 1,4-DHP derivative **1** were with an average diameter of 395 nm for the freshly prepared samples and 346 nm after storage, and PDI values were 0.342 and 0.305, respectively. Similar observations could be made for compound **5b** with 4-methylpyridinium substituents—it formed nanoparticles with the average diameter of 529 nm for the freshly prepared samples and 430 nm after storage. PDI values were 0.521 and 0.443, respectively. Nanoparticles of compound **5c** with 4-dimethylaminopyridinium substituents possessed an average diameter of 714 nm for the freshly prepared samples and increased to 458 nm after storage, with PDI values of 0.414 and 0.445, respectively. The mentioned values of PDI confirmed that the sample possesses average homogeneity. In the case of compound **5a**, the freshly prepared sample was more homogenous with a PDI value of 0.116, while the average diameter of nanoparticles was 781 nm. After the storage, the average diameter of nanoparticles increased to 873 nm, but the PDI value increased to 0.391. Nanoparticles of compound **5e** with 4-propylpyridinium substituents possessed an average diameter of 462 nm for the freshly prepared samples and decreased to 1003 nm after storage. In both cases samples were homogenous, which was confirmed by PDI values of 0.251 and 0.163, respectively.

The two-fold increase of particle size for comp. **5e** could be explained by the aggregation of particles or Ostwald ripening. A similar phenomena was also observed and described by other research groups [36,37]. It was demonstrated that the parameters of lipoplexes formed by 1,4-DHP amphiphiles **1**, **5a**,**b** did not differ much after one month storage at 4 °C. These data show that the samples were stable during 1 month of storage.

The ability to form nanoparticles by the amphiphilic 1,4-DHP is highly influenced by the substituents of its ring, which can be observed in our previous studies for the structurally related compounds. Thus, particles formed by 1,4-DHP amphiphile 1 had an average diameter of 110 nm and a PDI value of 0.345 [17], and particles formed by 1,1′-{[3,5-bis(dodecyloxycarbonyl)-4-(naphthalen-2-yl)-1,4-dihydropyridine-2,6-diyl]bis(methylene)}bis{4-[(E)-2-(naphthalen-2-yl)vinyl]pyridin-1-ium} dibromide had an average diameter of approximately 300 nm and a PDI value of 0.491 for the freshly prepared sample [24]. According to the literature, the size and homogeneity of samples also depend on the preparation method. It is underlined that, using the ethanol injection method, the concentration of ethanol should not exceed 7.5% in order to prevent liposome destabilization [38,39]. The other main drawback of ethanol injection method is connected with the final formation of a heterogeneous population of liposomes [40].

#### 3.3.2. Characterization of Lipoplexes

**Figure 3:**
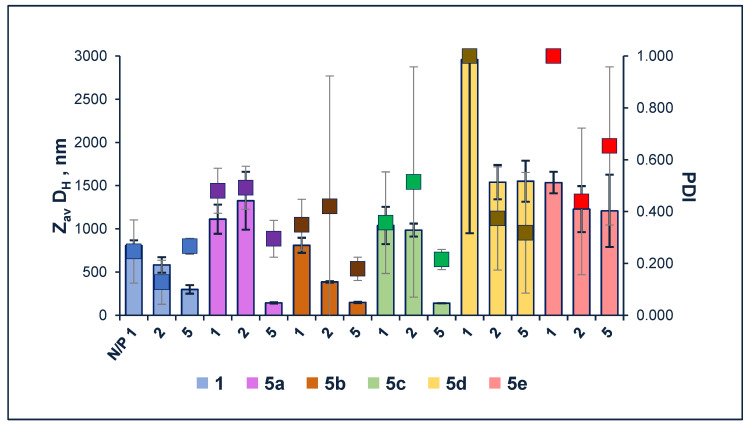
Values of Z-average (Z_av_D_H_) diameter and polydispersity index (PDI) of lipoplexes formed by 1,4-DHP amphiphiles **1** and **5** and mRNA at N/P charge ratios of 1, 2, and 5 obtained by DLS measurements. Error bars smaller than data point size are not visible.

The lipoplexes were prepared by injecting an ethanol solution of 1,4-DHP amphiphiles **1** and **5** into a solution containing a constant amount of mRNA at different N/P (nitrogen/phosphate) charge ratios. After the incubation, a constant amount of MEM was added to each sample of lipoplexes, followed by vortexing and incubation for 30 min prior to DLS measurements. The obtained data are summarized in Figure 3 (data from Table S2, Supplementary Materials). These measurements were performed for comparison of the sizes of empty liposomes and lipoplexes formed by lipids and mRNA.

The obtained data (Figure 3) demonstrate that the average diameters of lipoplexes formed by compounds **5d** and **5e** at all the tested N/P ratios were above 1200 nm. Thus, in the case of compound **5d** at N/P ratio 1, the average diameter was almost 3000 nm. PDI values of for lipoplexes of compounds **5d** and **5e** at N/P ratio of 1 were around 1, while at a N/P ratio of 2, PDI values were 0.373 and 0.439 and at N/P ratio of 5–0.318 or 0.653, respectively. The average diameter of lipoplexes formed by compounds **5a** and **5c** at N/P ratios of 1 and 2 were around 1000 nm with PDI values in the range of 0.357–0.514, but at a N/P ratio of 5, the average diameter of lipoplexes decreased to 143 and 139 nm with PDI values of 0.295 and 0.215, respectively. The average diameter of lipoplexes formed by compounds **1** and **5b** at N/P ratio of 1 were around 800 nm with PDI values of 0.246 and 0.349, a N/P ratio of 2–582 nm and 386 nm, and PDI values of 0.127 and 0.420, but a N/P ratio of 5–298 nm and 148 nm with PDI values of 0.266 and 0.179. This is in agreement with our previous observations—that the mean diameters of the complexes of structurally related 1,4-DHP amphiphiles with DNA at high N/P (4) are rather small (around 150 nm), while decreasing the N/P charge ratio increases the size of lipoplexes 10–35-fold [16].

The comparison of empty liposome parameters with lipolpexe parameters demonstrated that the size of lipoplexes at N/P ratios of 1 (Table S1, column 3 versus Table S2, column 3) slightly increased for comp. **5d**, 1.5 times for comp. **5a–c**, 2 times for comp. **1**, and 3 times for comp. **5e**. The size of lipoplexes at N/P ratios of 2 (Table S1, column 3 versus Table S2, column 5) decreased 1.4 and 1.7 times for comp. **5c** and **5d**, respectively, and increased 1.4–1.7 times for comp. **5c**, **1** and **5a**, as well as 2.5 times for comp. **5e**. The size of lipoplexes at N/P ratios of 5 (Table S1, column 3 versus Table S2, column 7) decreased 5 and 5.5 times for comp. **5c** and **5a**, respectively, 3.6 times for comp. **5b**, and 1.3 and 1.7 times for comp. **1** and **5d**, respectively, and increased 2.6 times for comp. **5e**.

During the determination of the size of liposome complexes formed by compounds with RNA at low N/P charge ratios, we observed larger particles and higher PDI values. We explain this by the fact that in order to compensate for all the negative charges on the RNA molecule with the charge of cations in the lipid molecule, the lipid molecules are oriented with the cation towards the RNA. Therefore, when such a complex is formed, its surface becomes hydrophobic, and the aggregation of these complexes occurs in the water media.

Our previous data demonstrated that the cytotoxicity of various N-unsubstituted 1,4-DHP amphiphile–pDNA complexes were in the same range as those determined for PEI 25 and DOTAP complexes at low N/P ratios (1 and 2), as more than 80% of the cells were found to be viable. At a N/P ratio of 4, most of the complexes became more cytotoxic. Cytotoxicity was further increased at a charge ratio of 8 [17]. Additionally, it was demonstrated that the transfection ability and cytotoxicity of 1,4-DHP amphiphiles was dependent on the compound structure, and showed different effects on the tested cell lines [20].

Taking the high similarity of the structures of compounds and cytotoxicity data from previous studies, parameters of formed nanoparticles and an analysis of results allow us to assume that lipoplexes formed by 1,4-DHP amphiphiles **1** and **5a**–**c** at N/P charge ratio of 5 meet the requirements of prospective carrier system parameters.

## 4. Conclusions

Novel *N*-benzyl 1,4-DHP amphiphiles as prospective delivery systems were successfully obtained and characterized. The variations in the structure of the original *N*-benzyl 1,4-DHP amphiphiles **5** were performed by the introduction of the benzyl substituent at N atom of 1,4-DHP cycle and by the variation of pyridinium substituents of 1,4-DHP cycle. Studies of monolayers composed of 1,4-DHP amphiphiles revealed that the mean molecular areas of the compounds extracted from the π−A isotherms and demonstrated that MMA values depend on the amphiphile structure. The introduction of *N*-benzyl substituent at the position 1 of 1,4-DHP ring enlarged the mean molecular area by almost half. All the formed monolayers collapsed in the liquid-condensed phase. 

Nanoparticles obtained by the ethanol injection method possessed positive surface charge with an average diameter of 395–2570 nm for the freshly prepared samples and 346–1195 nm for the samples after one month of storage at 4 °C. Liposomes formed by 1,4-DHP amphiphiles **1**, **5a**,**b** were stable after 1 month of storage. It was confirmed that the structure of the cationic head-group affects the size of the formed nanoparticles. Thus, *N*-benzyl substitution at the 1,4-DHP cycle, in combination with the substituents at the pyridinium moieties, has a negative impact on the nanoparticle homogeneity and stability during storage. The diameters of lipoplexes formed by 1,4-DHP amphiphiles and mRNA at N/P charge ratios of 1, 2, and 5 were in the range of 139–2959 nm and were related to the structure of compounds and the N/P charge ratio. The preliminary results suggest that the lipoplexes formed by mRNA and 1,4-DHP amphiphiles **1** and **5a–c** at N/P charge ratio 5 are prospective. The results of this study suggest that 1,4-DHP amphiphiles **1** and **5a–c** have favorable properties for potential applications in nanomedicine as DNA or RNA delivery agents.

## Figures and Tables

**Table 1:** Mechanical properties of the monolayers composed of 1,4-DHP amphiphiles **1** and **5** obtained using the Langmuir–Blodgett trough, concentration of samples: 1 mg/mL; for comp. **5a**—0.125 mg/mL; subphase: deionized H_2_O. C_s_^−1^ (mN/m) is the maximum value of compressibility modulus; P (mN/m) is the surface pressure of the monolayers at C_s_^−1^_max_; MMA (Å^2^) is the mean molecular area.

Entry	Comp.	Cs^−1^, mN/m	P, mN/m	MMA, Å^2^
1	**1**	169.64 ± 2.05	24.61 ± 0.71	96.05 ± 4.69
2	5a *	211.94 ± 20.35	37.93 ± 1.44	138.11 ± 3.57
3	**5b**	154.67 ± 1.88	27.74 ± 0.63	132.58 ± 4.90
4	**5c**	201.03 ± 33.56	35.61 ± 1.22	131.15 ± 7.15
5	5d *	167.73 ± 0.42	36.05 ± 1.19	139.06 ± 1.94
6	**5e ^#^**	146.16 ± 20.59	36.02 ± 0.54	191.26 ± 8.21
		102.16 ± 25.46	44.05 ± 1.36	
		142.22 ± 7.45	54.94 ± 1.33	

* 0.01 M KBr solution. ^#^ π−A isotherm with 3 zones.

**Table 2:** Values of the zeta-potentials (Z_pot_) and critical aggregation concentration (CAC) of nanoparticles formed by 1,4-DHP amphiphiles **1** and **5** in an aqueous solution obtained by DLS measurements. The Z_pot_ value gives information about the surface charge of the nanoparticles and the CAC value characterizes the concentration above which vesicles are formed.

Entry	Value	Comp. 1	Comp. 5a	Comp. 5b	Comp. 5c	Comp. 5d	Comp. 5e
1	Z_pot_, mV	75.4 ± 7.5	40.6 ± 0.9	89.0 ± 1.1	56.8 ± 0.4	35.7 ± 2.5	90.9 ± 9.1
2	CAC, µM	1.06	0.46	1.16	0.01	0.95	2.65

## Data Availability

Not applicable.

## Supplementary Materials

The following supporting information can be downloaded at: https://www.mdpi.com/article/10.3390/ma16124206/s1. The following supporting information is available online: ^1^H NMR, ^13^C NMR and HRMS spectra of compounds **2**, **3** and **5a**–**e** (^1^H NMR (Figures S1, S4, S7, S10, S13, S16 and S19), ^13^C NMR (Figures S2, S5, S8, S11, S14, S17 and S20) and HRMS spectra (Figures S3, S6, S9, S12, S15, S18 and S21) of compounds **2**, **3** and **5a**–**e**, surface pressure—mean molecular area isotherms of 1,4-DHP amphiphiles **1** and **5** (Figure S22) and compressibility modulus-surface pressure dependences graphs of 1,4-DHP amphiphiles **1** and **5** (Figure S23). Table S1 with DLS data for nanoparticles obtained from 1,4-DHP amphiphiles **1** and **5** and Table S2 with DLS data for lipolexes formed by nanoparticles from 1,4-DHP amphiphiles **1** and **5** nanoparticles and mRNA.
