# Supplementary material for: Synthesis and Characterization of Novel Amphiphilic N-Benzyl 1,4-Dihydropyridine Derivatives—Evaluation of Lipid Monolayer and Self-Assembling Properties
Source: Materials (Basel). 2023 Jun 6;16(12):4206. doi: 10.3390/ma16124206 (PMC10301244; doi:10.3390/ma16124206)
Supplement: Supplementary file 1 [file materials-16-04206-s001.zip › materials-2359767-supplementary.pdf]

Supplementary Materials

Synthesis and characterization of novel amphiphilic *N*-benzyl 1,4-dihydropyridine derivatives – evaluation of lipid monolayer and self-assembling properties

Anna Krapivina[1], Davis Lacis[2,3], Martins Rucins[2], Mara Plotniece[1,3], Karlis Pajuste[2], Arkadij Sobolev[2], Aiva Plotniece[1,2,*]

[1] Department of Pharmaceutical Chemistry, Faculty of Pharmacy, Riga Stradiņš University, Dzirciema 16, Riga LV-1007, Latvia; akrapivina17@gmail.com (A.K.), mara.plotniece@rsu.lv (M.P.)
[2] Latvian Institute of Organic Synthesis, Aizkraukles 21, Riga LV-1006, Latvia; davis.lacis@osi.lv (D.L.), kpajuste@osi.lv (K.P.), arkady@osi.lv (A.S.), aiva@osi.lv (A.P)
[3] Faculty of Materials Science and Applied Chemistry, Riga Technical University, P. Valdena 3, Riga LV-1048, Latvia
* Correspondence: aiva@osi.lv; Tel.: + 37167014852

Table of Contents

Figure S1. ^{1}H NMR spectrum of didodecyl 1-benzyl-2,6-dimethyl-4-phenyl-1,4-dihydropyridine-3,5-dicarboxylate (**2**) 3
Figure S2. ^{13}C NMR spectrum of didodecyl 1-benzyl-2,6-dimethyl-4-phenyl-1,4-dihydropyridine-3,5-dicarboxylate (**2**) 3
Figure S3. HRMS data of 1 didodecyl 1-benzyl-2,6-dimethyl-4-phenyl-1,4-dihydropyridine-3,5-dicarboxylate (**2**) 4
Figure S4. ^{1}H NMR spectrum of didodecyl 1-benzyl-2,6-bis(bromomethyl)-4-phenyl-1,4-dihydro-pyridine-3,5-dicarboxylate (**3**) 4
Figure S5. ^{13}C NMR spectrum of didodecyl 1-benzyl-2,6-bis(bromomethyl)-4-phenyl-1,4-dihydropyridine-3,5-dicarboxylate (**3**) 5
Figure S6. HRMS data of didodecyl 1-benzyl-2,6-bis(bromomethyl)-4-phenyl-1,4-dihydropyridine-3,5-dicarboxylate (**3**) 5
Figure S7. ^{1}H NMR spectrum of 1,1'-((1-benzyl-3,5-bis((dodecyloxy)carbonyl)-4-phenyl-1,4-dihydro-pyridine-2,6-diyl)bis(methylene))bis(pyridin-1-ium) dibromide (**5a**) 6
Figure S8. ^{13}C NMR spectrum of 1,1'-((1-benzyl-3,5-bis((dodecyloxy)carbonyl)-4-phenyl-1,4-dihydropyridine-2,6-diyl)bis(methylene))bis(pyridin-1-ium) dibromide (**5a**) 6
Figure S9. HRMS data of 1,1'-((1-benzyl-3,5-bis((dodecyloxy)carbonyl)-4-phenyl-1,4-dihydropyridine-2,6-diyl)bis(methylene))bis(pyridin-1-ium) dibromide (**5a**) 7
Figure S10. ^{1}H NMR spectrum of 1,1'-((1-benzyl-3,5-bis((dodecyloxy)car-bonyl)-4-phenyl-1,4-dihydropyridine-2,6-diyl)bis(methylene))bis(pyridin-1-ium) dibromide (**5b**) 7
Figure S11. ^{13}C NMR spectrum of 1,1'-((1-benzyl-3,5-bis((dodecyloxy)car-bonyl)-4-phenyl-1,4-dihydropyridine-2,6-diyl)bis(methylene))bis(pyridin-1-ium) dibromide (**5b**) 8
Figure S12. HRMS data of 1,1'-((1-benzyl-3,5-bis((dodecyloxy)carbonyl)-4-phenyl-1,4-dihydropyridine-2,6-diyl)bis(methylene))bis(pyridin-1-ium) dibromide (**5b**) 8
Figure S13. ^{1}H NMR spectrum of 1,1'-((1-benzyl-3,5-bis((dodecyl-oxy)carbonyl)-4-phenyl-1,4-dihydropyridine-2,6-diyl)bis(methylene))bis(4-(dimethylamino)pyridin-1-ium) (**5c**) 9
Figure S14. ^{13}C NMR spectrum of 1,1'-((1-benzyl-3,5-bis((dodecyloxy)car-bonyl)-4-phenyl-1,4-dihydropyridine-2,6-diyl)bis(methylene))bis(4-(dimet-hylamino)pyridin-1-ium) (**5c**) 9

Figure S15. HRMS data of 1,1'-((1-benzyl-3,5-bis((dodecyloxy)carbonyl)-4-phenyl-1,4-dihydropyridine-2,6-diyl)bis(methylene))bis(4-(dimethylamino)pyridin-1-ium) (**5c**) 10
Figure S16. ^{1}H NMR spectrum of 1,1'-((1-benzyl-3,5-bis((dodecyloxy)carbonyl)-4-phenyl-1,4-dihydro-pyridine-2,6-diyl)bis(methylene))bis(4-phenylpyridin-1-ium) (**5d**) 10
Figure S17. ^{13}C NMR spectrum of 1,1'-((1-benzyl-3,5-bis((dodecyloxy)carbonyl)-4-phenyl-1,4-dihydro-pyridine-2,6-diyl)bis(methylene))bis(4-phenylpyridin-1-ium) (**5d**) 11
Figure S18. HRMS data of 1,1'-((1-benzyl-3,5-bis((dodecyloxy)carbonyl)-4-phenyl-1,4-dihydropyridine-2,6-diyl)bis(methylene))bis(4-phenylpyridin-1-ium) (**5d**) 11
Figure S19. ^{1}H NMR spectrum of 1,1'-((1-benzyl-3,5-bis((dodecyloxy)carbonyl)-4-phenyl-1,4-dihydro-pyridine-2,6-diyl)bis(methylene))bis(4-propylpyridin-1-ium) (**5e**) 12
Figure S20. ^{13}C NMR spectrum of 1,1'-((1-benzyl-3,5-bis((dodecyloxy)carbonyl)-4-phenyl-1,4-dihydro-pyridine-2,6-diyl)bis(methylene))bis(4-propylpyridin-1-ium) (**5e**) 12
Figure S21. HRMS data of 1,1'-((1-benzyl-3,5-bis((dodecyloxy)carbonyl)-4-phenyl-1,4-dihydropyridine-2,6-diyl)bis(methylene))bis(4-propylpyridin-1-ium) (**5e**) 13
Figure S22. Surface pressure – mean molecular area isotherms of tested 1,4-DHP amphiphiles **1** and **5** at 25±1°C 14
Figure S23. Compressibility modulus-surface pressure dependences obtained for the 1,4-DHP amphiphiles **1** and **5** monolayers 14
Table S1. Values of Z-average ($Z_{av}D_H$) diameter and polydispersity index (PDI) of nanoparticles formed by 1,4-DHP amphiphiles **1** and **5** at the 0.1mM concentration obtained by DLS measurements for freshly prepared samples and after 1 month storage. The PDI value describes polydispersity of the sample; the Z_{av} diameter represents the average hydrodynamic diameter of all nanoparticles in the sample 15
Table S2. Values of Z-average ($Z_{av}D_H$) diameter and polydispersity index (PDI) of lipoplexes formed by 1,4-DHP amphiphiles **1** and **5** and mRNA at different N/P charge ratios obtained by DLS measurements for freshly prepared samples. The PDI value describes polydispersity of the sample; the $Z_{av}D_H$ diameter represents the average hydrodynamic diameter of all nanoparticles in the sample 15

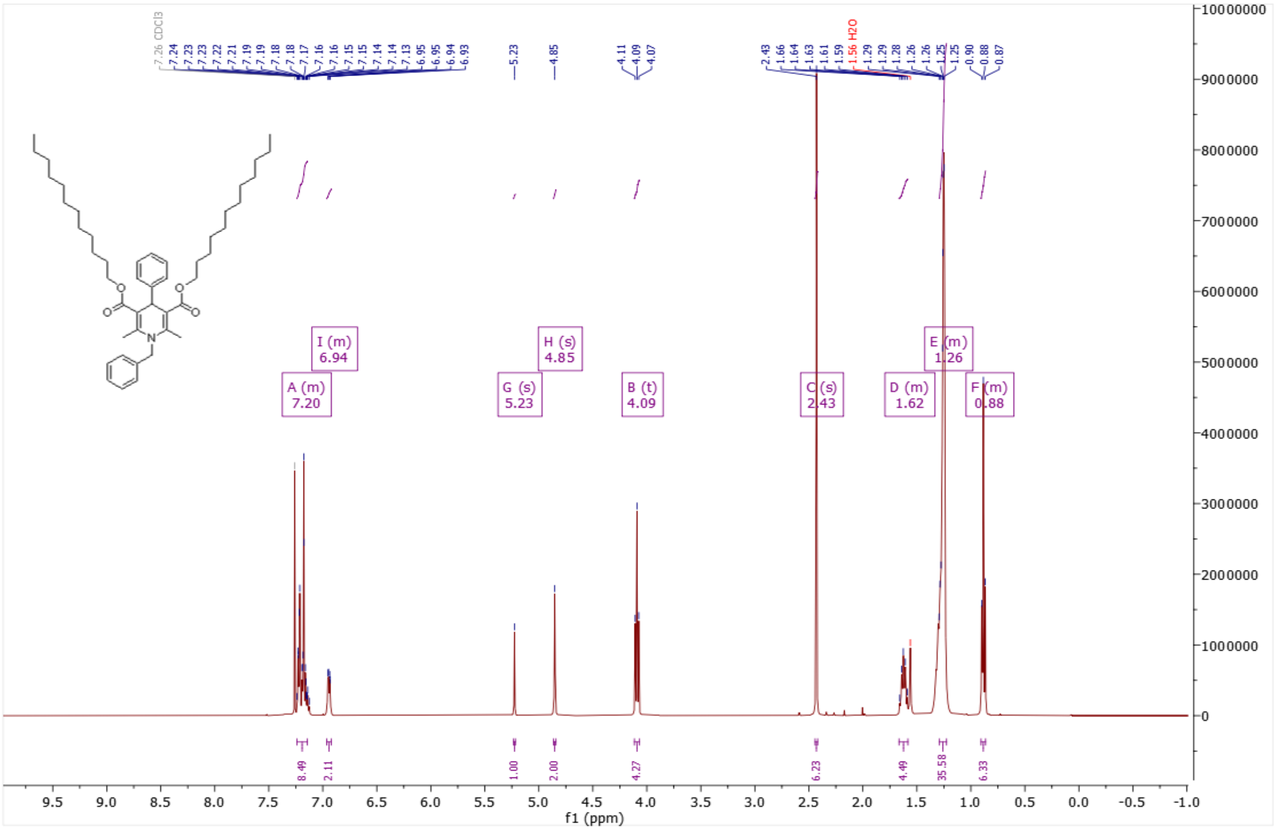

Figure S1. ^{1}H NMR spectrum of didodecyl 1-benzyl-2,6-dimethyl-4-phenyl-1,4-dihydropyridine-3,5-dicarboxylate (**2**).

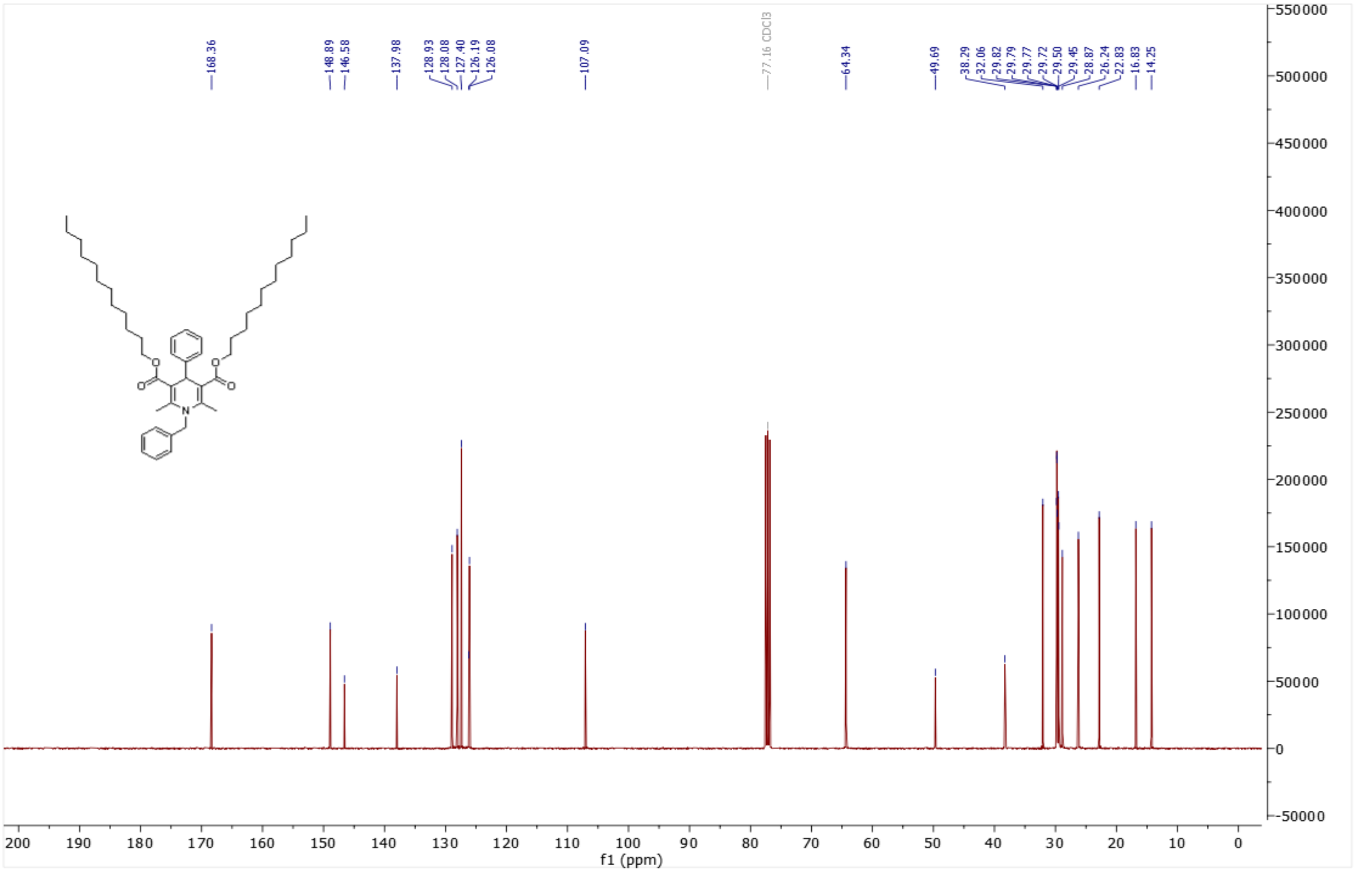

Figure S2. ^{13}C NMR spectrum of didodecyl 1-benzyl-2,6-dimethyl-4-phenyl-1,4-dihydropyridine-3,5-dicarboxylate (**2**)

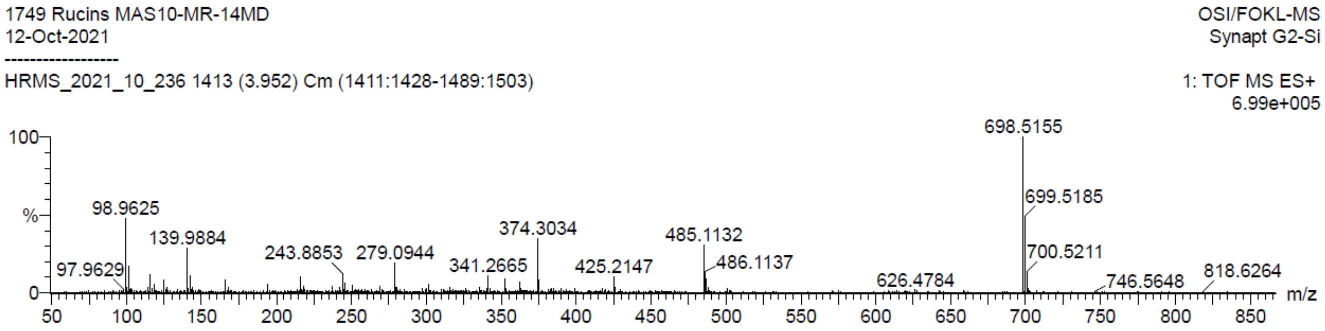

Minimum:	80.00				-1.5		
Maximum:	100.00		3.0	10.0	50.0		
Mass	RA	Calc. Mass	mDa	PPM	DBE	Conf(%)	Formula
698.5155	100.00	698.5148	0.7	1.0	13.5	90.34	C46 H68 N O4
		698.5162	-0.7	-1.0	18.5	9.66	C47 H64 N5

Figure S3. HRMS data of 1 didodecyl 1-benzyl-2,6-dimethyl-4-phenyl-1,4-dihydropyridine-3,5-dicarboxylate (**2**)

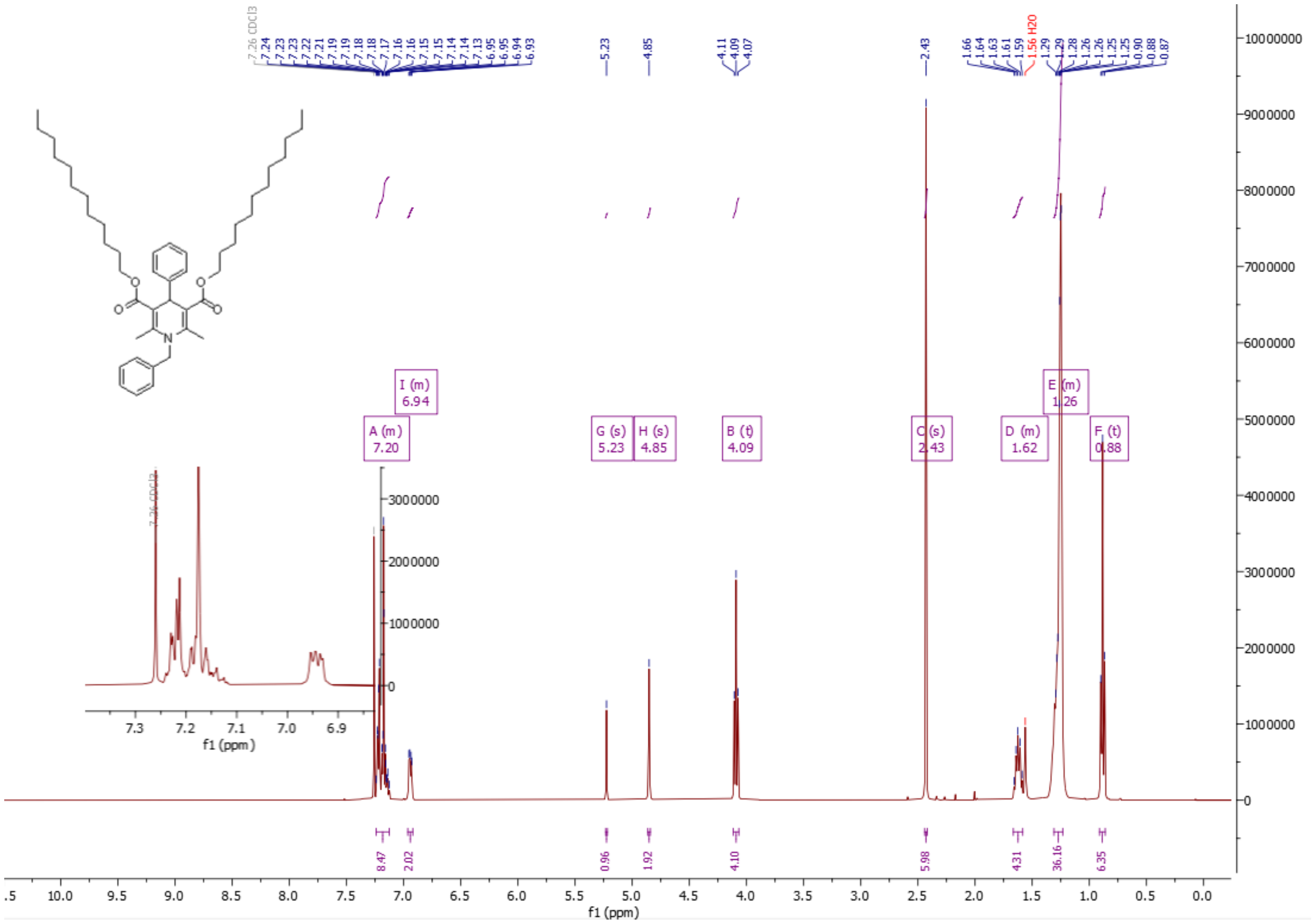

Figure S4. ^{1}H NMR spectrum of didodecyl 1-benzyl-2,6-bis(bromomethyl)-4-phenyl-1,4-dihydropyridine-3,5-dicarboxylate (**3**)

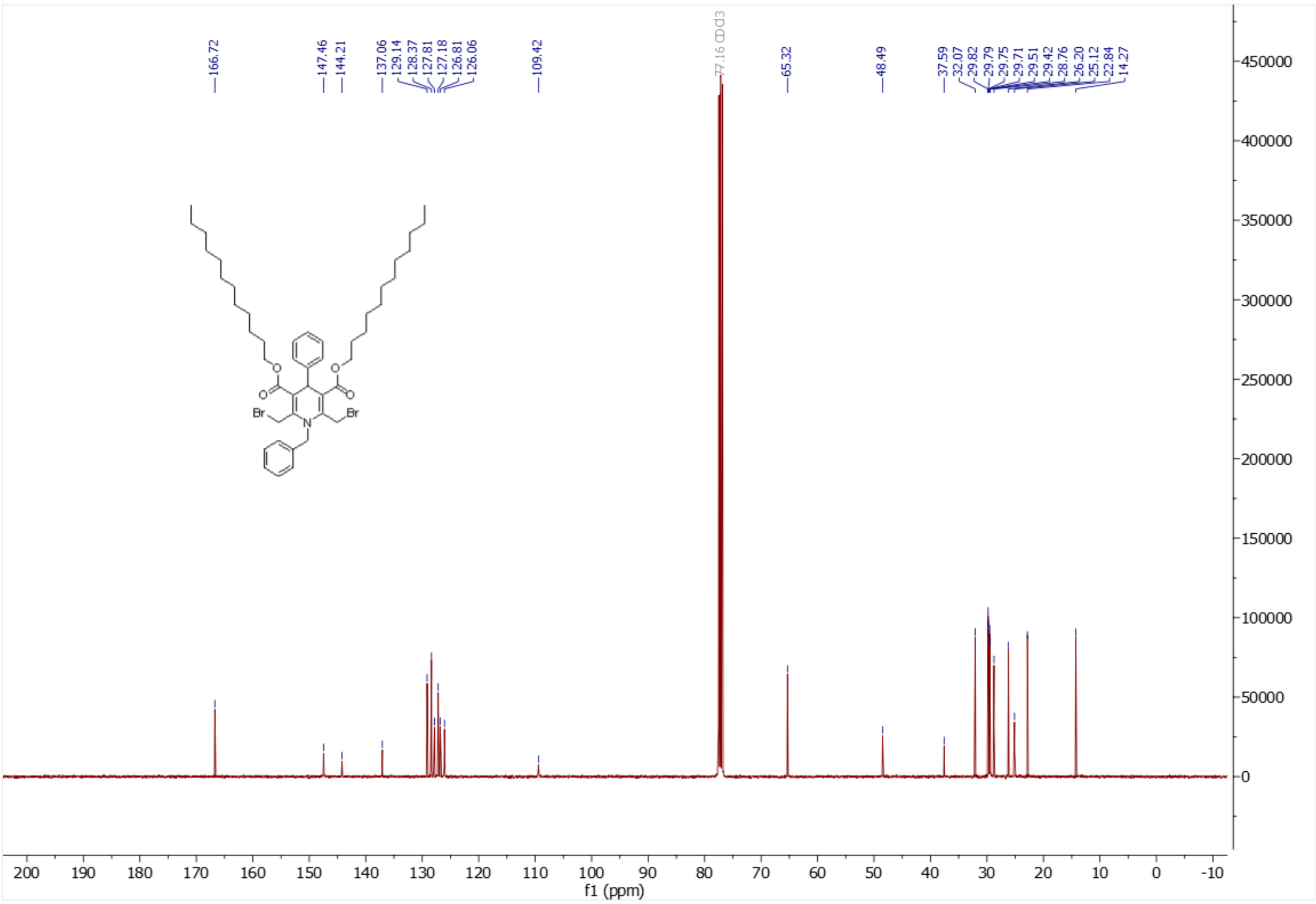

Figure S5. ^{13}C NMR spectrum of didodecyl 1-benzyl-2,6-bis(bromomethyl)-4-phenyl-1,4-dihydropyridine-3,5-dicarboxylate (**3**)

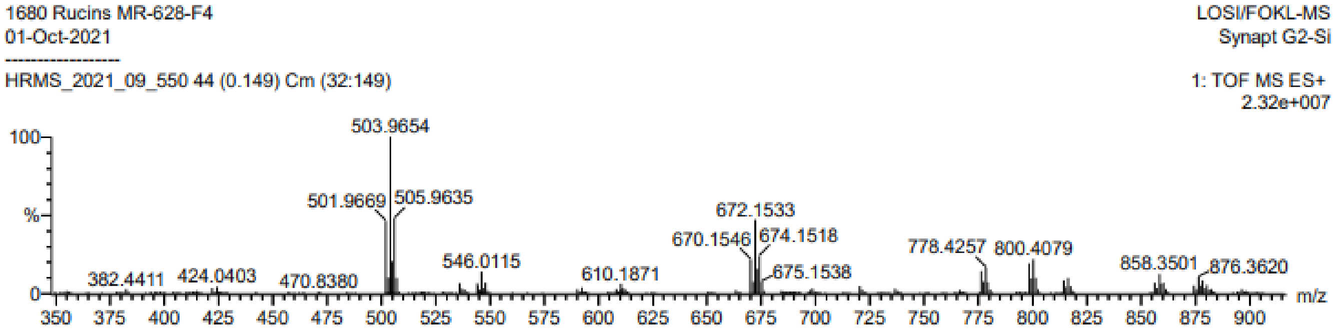

Minimum:				-1.5		
Maximum:		3.0	10.0	50.0		
Mass	Calc. Mass	mDa	PPM	DBE	Conf(%)	Formula
856.3491	856.3488	0.3	0.4	13.5	45.07	C42 H64 N7 O2 Br2
	856.3515	-2.4	-2.8	12.5	39.32	C46 H68 N O4 Br2
	856.3475	1.6	1.9	8.5	11.83	C41 H68 N3 O6 Br2
	856.3507	-1.6	-1.9	0.5	3.77	C30 H68 N9 O9 Br2

Figure S6. HRMS data of didodecyl 1-benzyl-2,6-bis(bromomethyl)-4-phenyl-1,4-dihydropyridine-3,5-dicarboxylate (**3**)

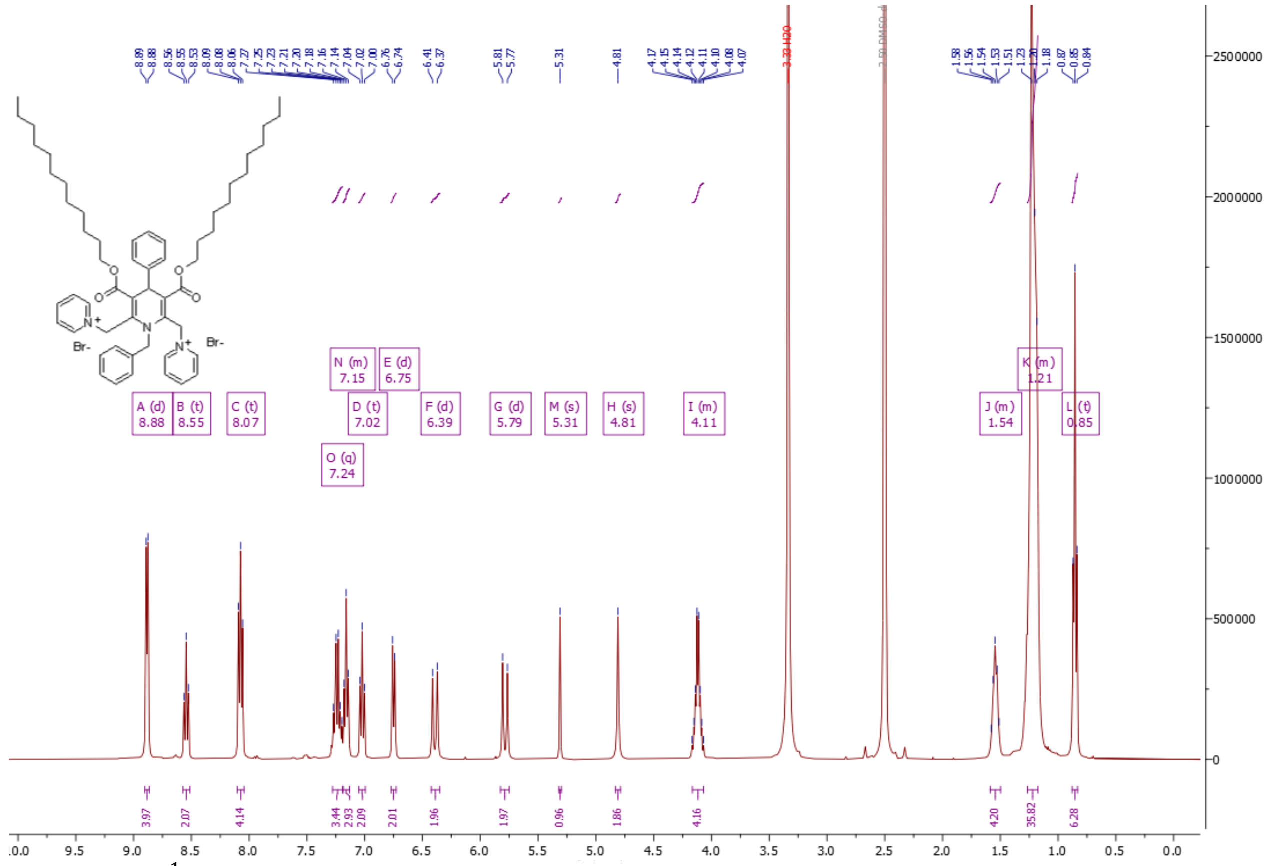

Figure S7. ^{1}H NMR spectrum of 1,1'-((1-benzyl-3,5-bis((dodecyloxy)carbonyl)-4-phenyl-1,4-dihydropyridine-2,6-diyl)bis(methylene))bis(pyridin-1-ium) dibromide (**5a**)

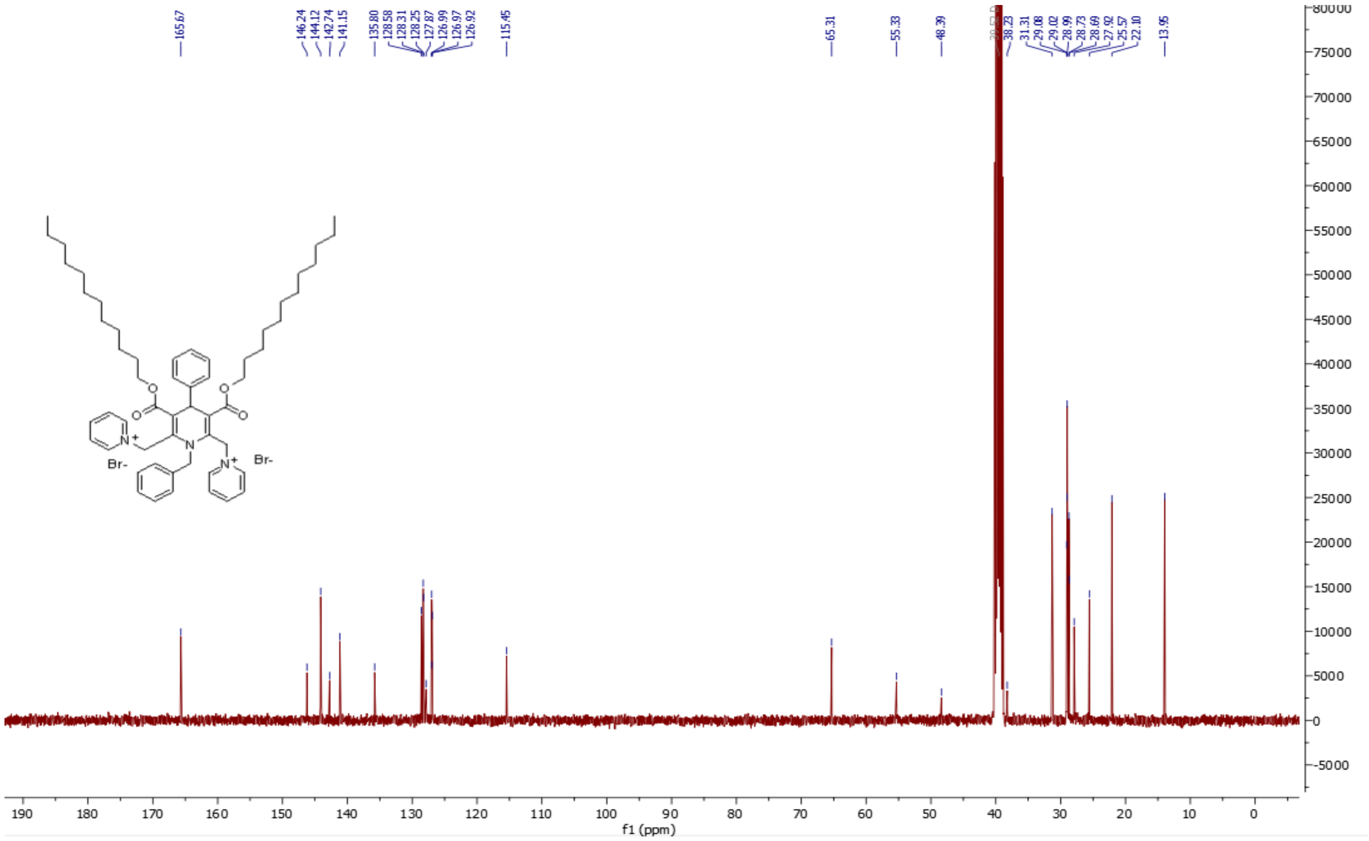

Figure S8. ^{13}C NMR spectrum of 1,1'-((1-benzyl-3,5-bis((dodecyloxy)carbonyl)-4-phenyl-1,4-dihydropyridine-2,6-diyl)bis(methylene))bis(pyridin-1-ium) dibromide (**5a**)

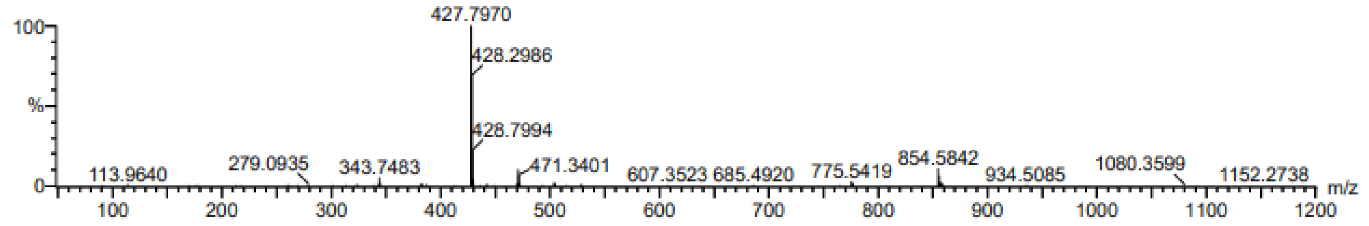

Minimum:				-1.5		
Maximum:		3.0	10.0	50.0		
Mass	Calc. Mass	mDa	PPM	DBE	Conf(%)	Formula
854.5842	854.5836	0.6	0.7	20.5	65.74	C56 H76 N3 O4
	854.5849	-0.7	-0.8	25.5	19.55	C57 H72 N7
	854.5868	-2.6	-3.0	12.5	5.85	C45 H76 N9 O7
	854.5854	-1.2	-1.4	7.5	5.07	C44 H80 N5 O11
	854.5841	0.1	0.1	2.5	3.79	C43 H84 N O15

Figure S9. HRMS data of 1,1'-((1-benzyl-3,5-bis((dodecyloxy)carbonyl)-4-phenyl-1,4-dihydropyridine-2,6-diyl)bis(methylene))bis(pyridin-1-ium) dibromide (**5a**)

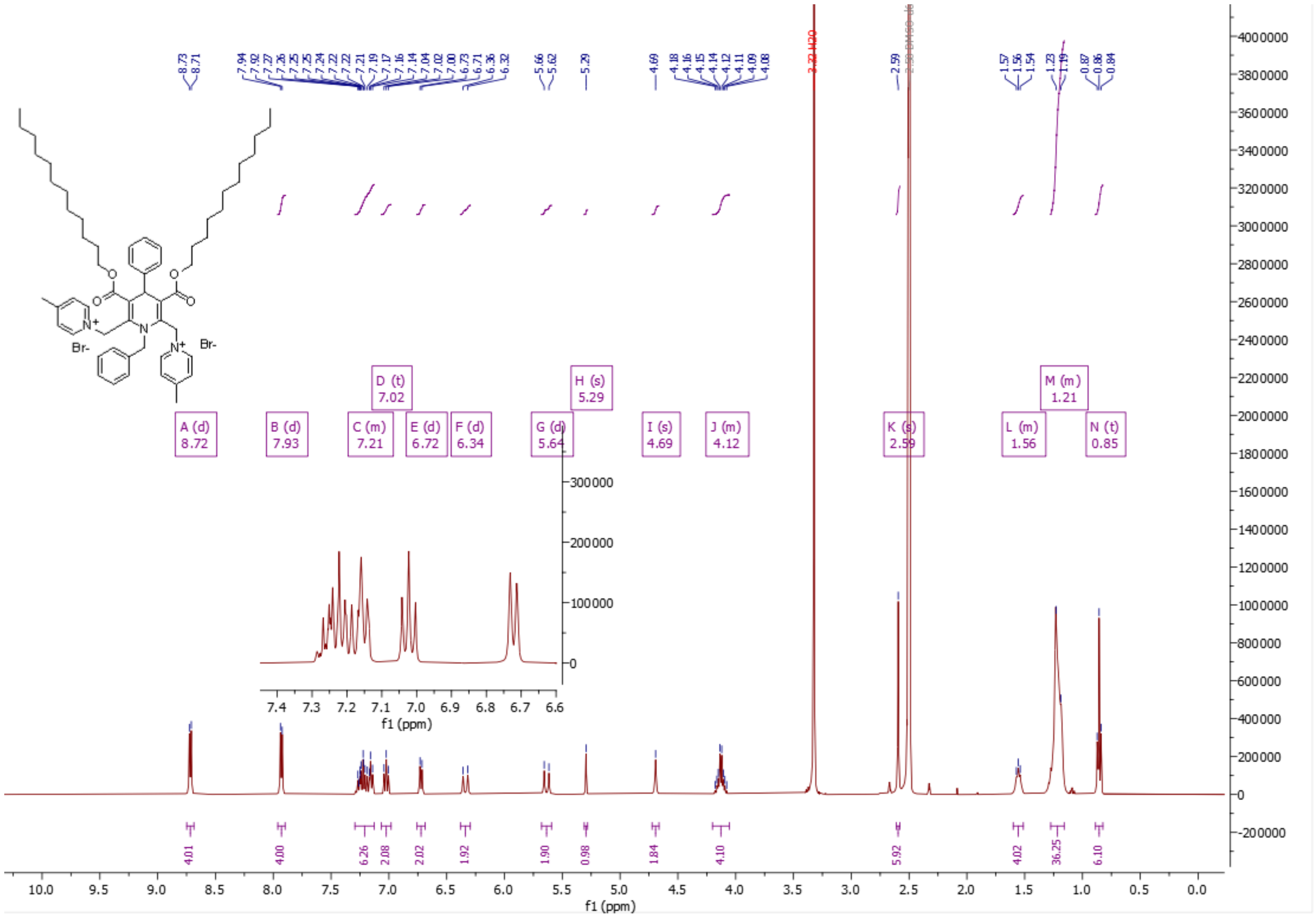

Figure S10. ^{1}H NMR spectrum of 1,1'-((1-benzyl-3,5-bis((dodecyloxy)carbonyl)-4-phenyl-1,4-dihydropyridine-2,6-diyl)bis(methylene))bis(pyridin-1-ium) dibromide (**5b**)

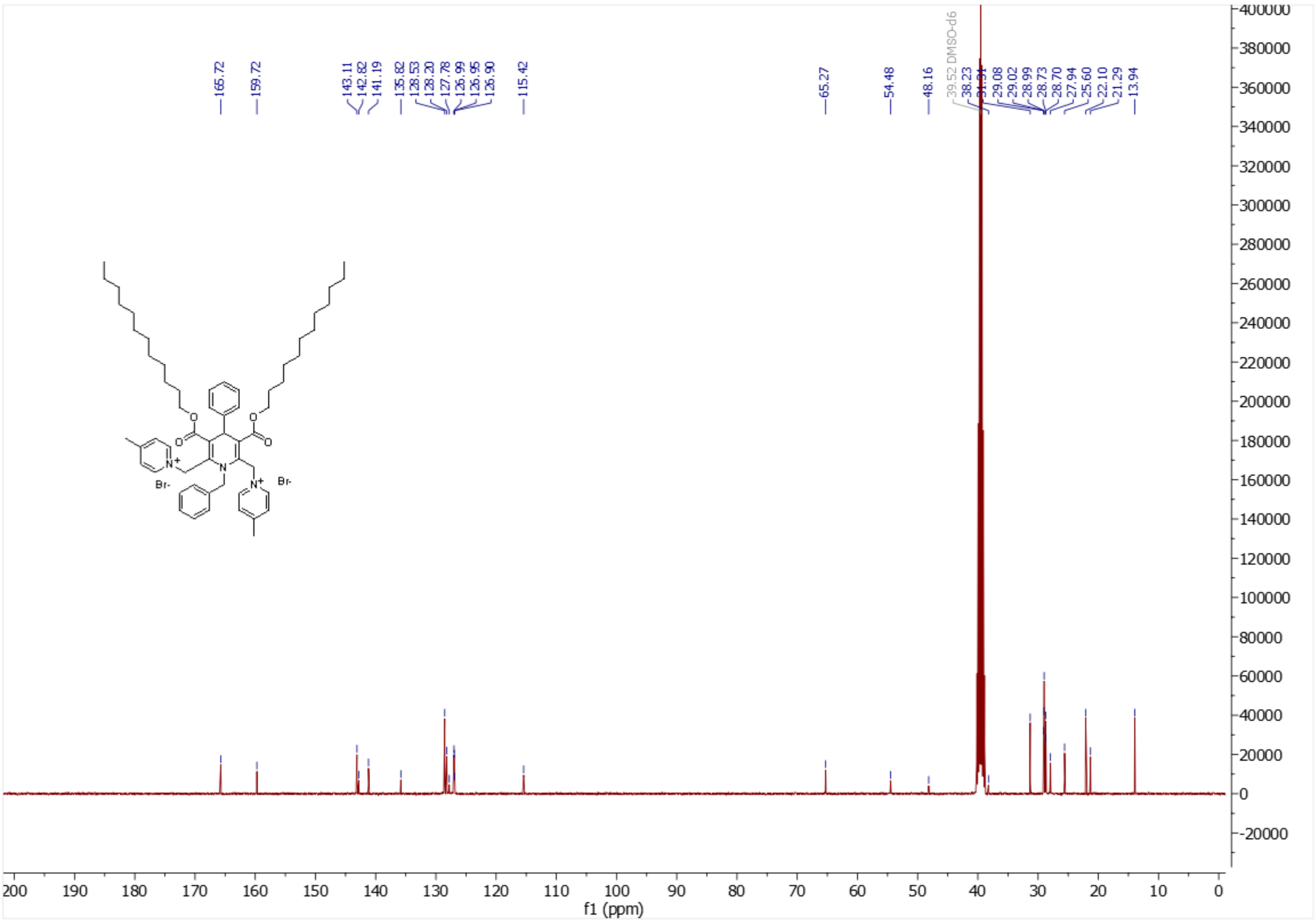

Figure S11. ^{13}C NMR spectrum of 1,1'-((1-benzyl-3,5-bis((dodecyloxy)carbonyl)-4-phenyl-1,4-dihydropyridine-2,6-diyl)bis(methylene))bis(pyridin-1-ium) dibromide (**5b**)

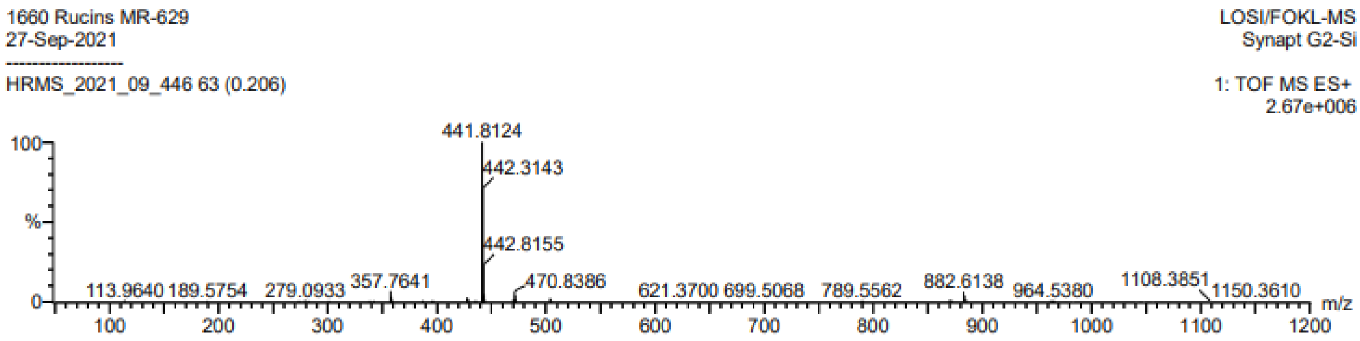

Minimum:				-1.5		
Maximum:		3.0	10.0	50.0		
Mass	Calc. Mass	mDa	PPM	DBE	Conf(%)	Formula
882.6138	882.6127	1.1	1.2	3.5	0.00	C41 H84 N7 O13
	882.6149	-1.1	-1.2	20.5	0.08	C58 H80 N3 O4
	882.6154	-1.6	-1.8	2.5	0.00	C45 H88 N O15
	882.6122	1.6	1.8	21.5	99.91	C54 H76 N9 O2
	882.6162	-2.4	-2.7	25.5	0.01	C59 H76 N7

Figure S12. HRMS data of 1,1'-((1-benzyl-3,5-bis((dodecyloxy)carbonyl)-4-phenyl-1,4-dihydropyridine-2,6-diyl)bis(methylene))bis(pyridin-1-ium) dibromide (**5b**)

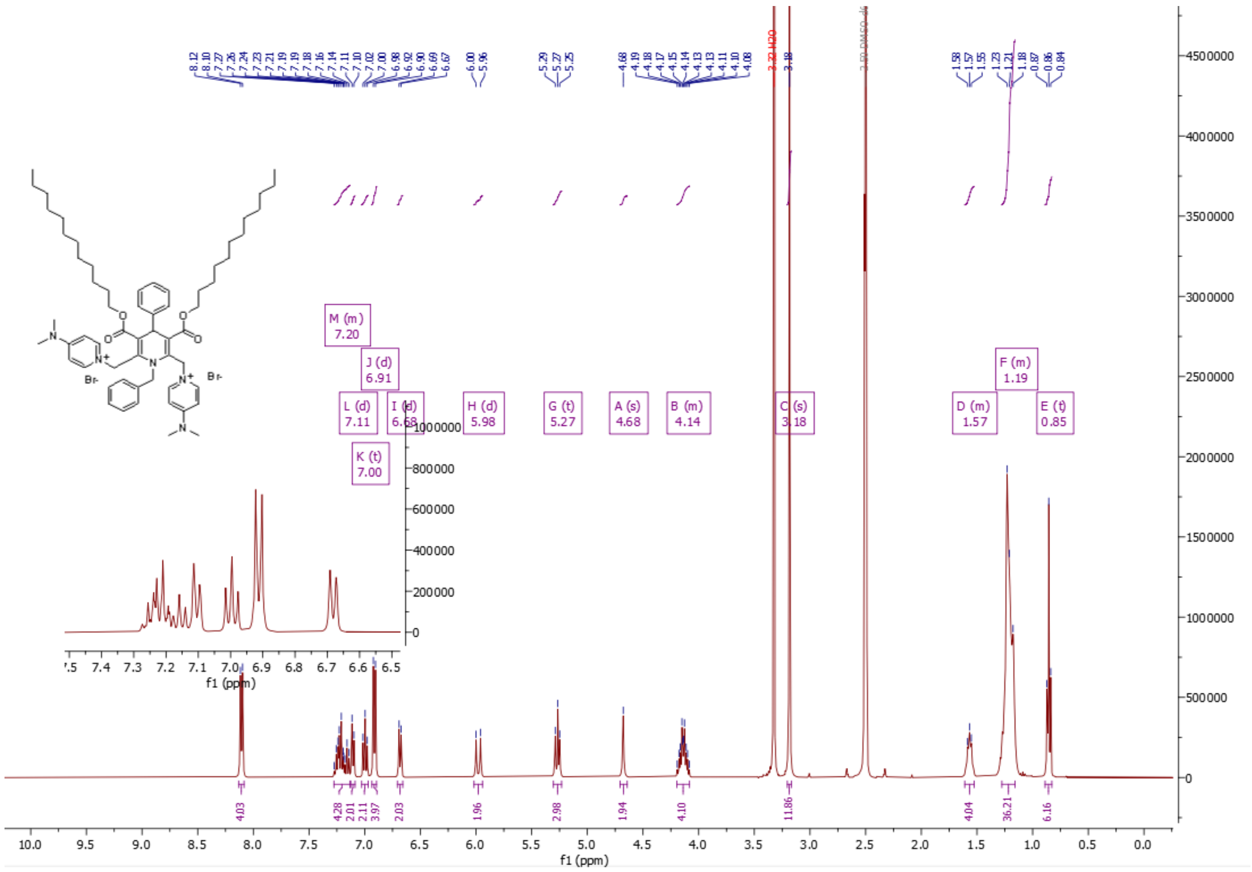

Figure S13. ^{1}H NMR spectrum of 1,1'-((1-benzyl-3,5-bis((dodecyloxy)carbonyl)-4-phenyl-1,4-dihydropyridine-2,6-diyl)bis(methylene))bis(4-(dimethylamino)pyridin-1-ium) (**5c**)

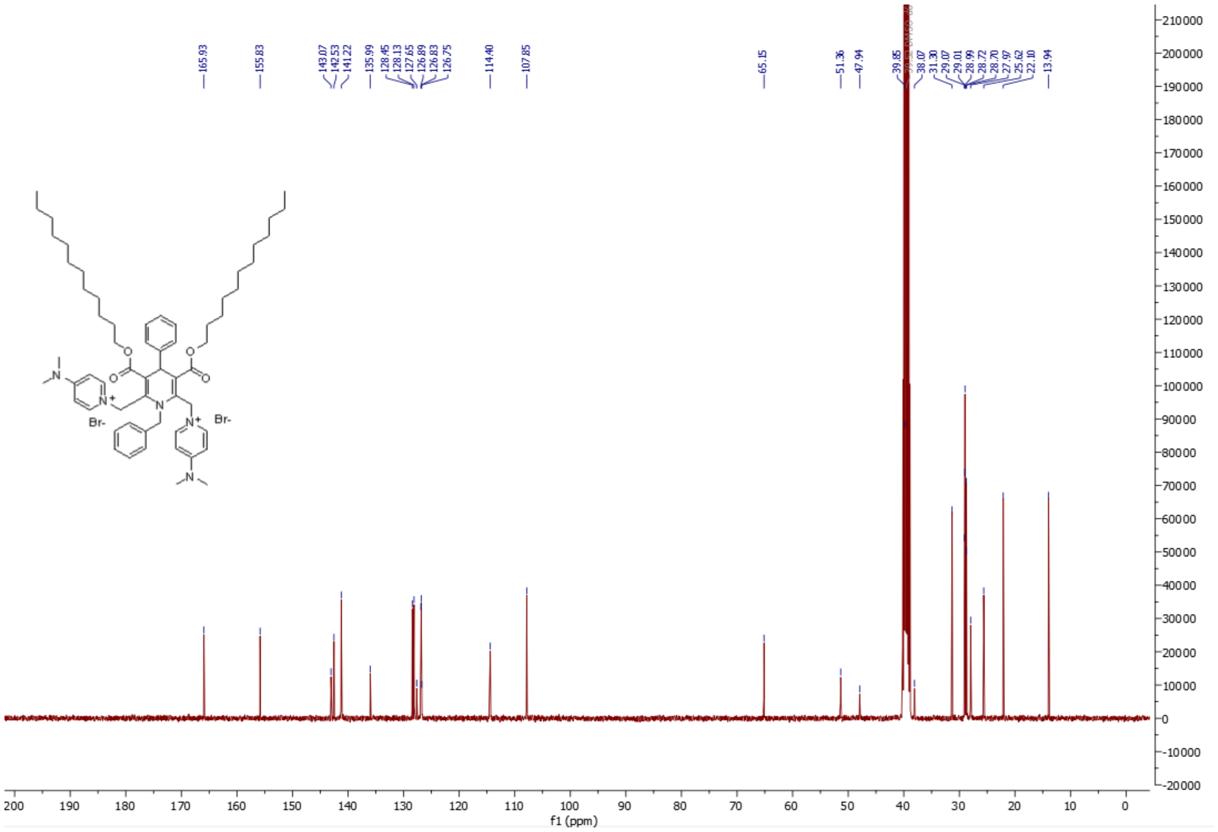

Figure S14. ^{13}C NMR spectrum of 1,1'-((1-benzyl-3,5-bis((dodecyloxy)carbonyl)-4-phenyl-1,4-dihydropyridine-2,6-diyl)bis(methylene))bis(4-(dimethylamino)pyridin-1-ium) (**5c**)

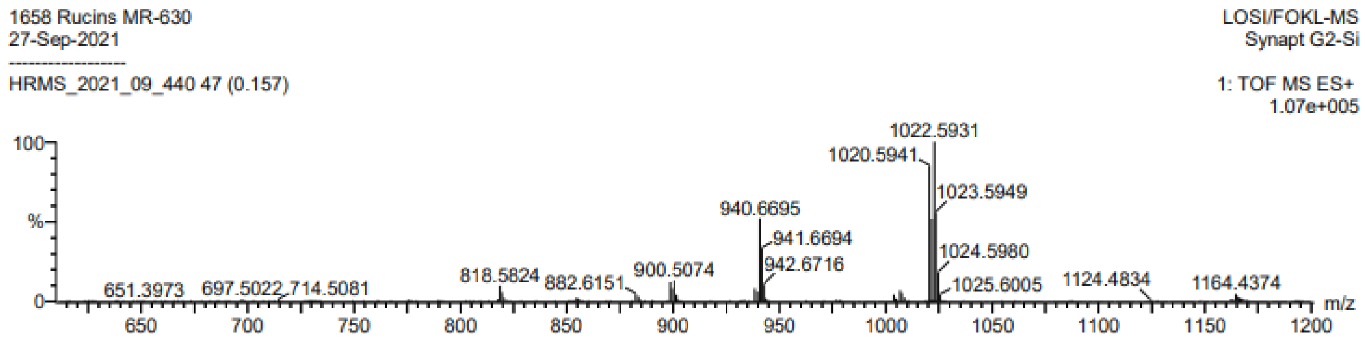

		mDa	PPM	DBE		
Minimum:				-1.5		
Maximum:		3.0	10.0	50.0		

Mass	Calc. Mass	mDa	PPM	DBE	Conf(%)	Formula
940.6695	940.6680	1.5	1.6	20.5	57.04	C60 H86 N5 O4
	940.6693	0.2	0.2	25.5	17.24	C61 H82 N9
	940.6698	-0.3	-0.3	7.5	16.39	C48 H90 N7 O11
	940.6685	1.0	1.1	2.5	8.31	C47 H94 N3 O15
	940.6720	-2.5	-2.7	24.5	1.01	C65 H86 N3 O2

Figure S15. HRMS data of 1,1'-((1-benzyl-3,5-bis((dodecyloxy)carbonyl)-4-phenyl-1,4-dihydropyridine-2,6-diyl)bis(methylene))bis(4-(dimethylamino)pyridin-1-ium) (**5c**)

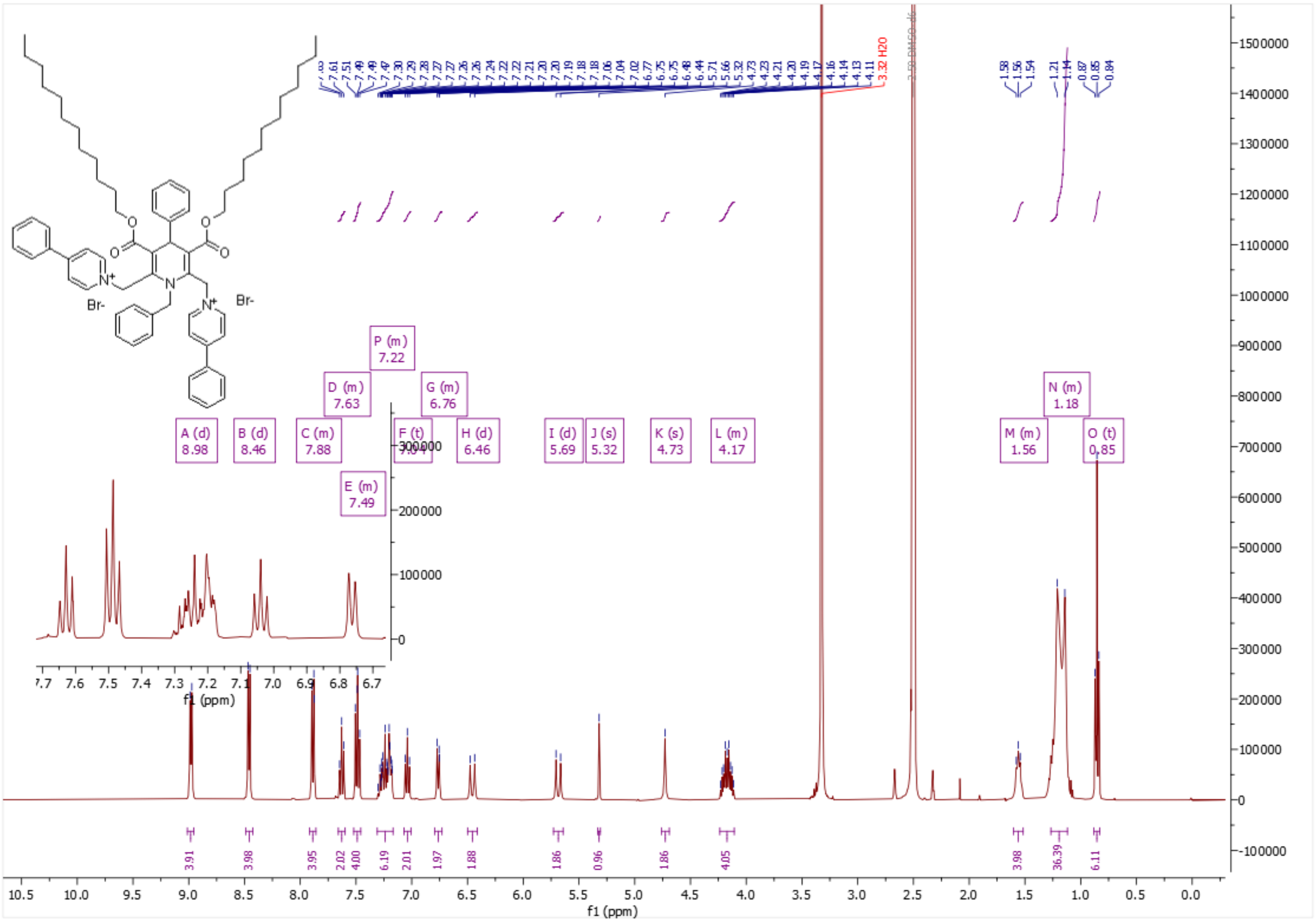

Figure S16. ^{1}H NMR spectrum of 1,1'-((1-benzyl-3,5-bis((dodecyloxy)carbonyl)-4-phenyl-1,4-dihydropyridine-2,6-diyl)bis(methylene))bis(4-phenylpyridin-1-ium) (**5d**)

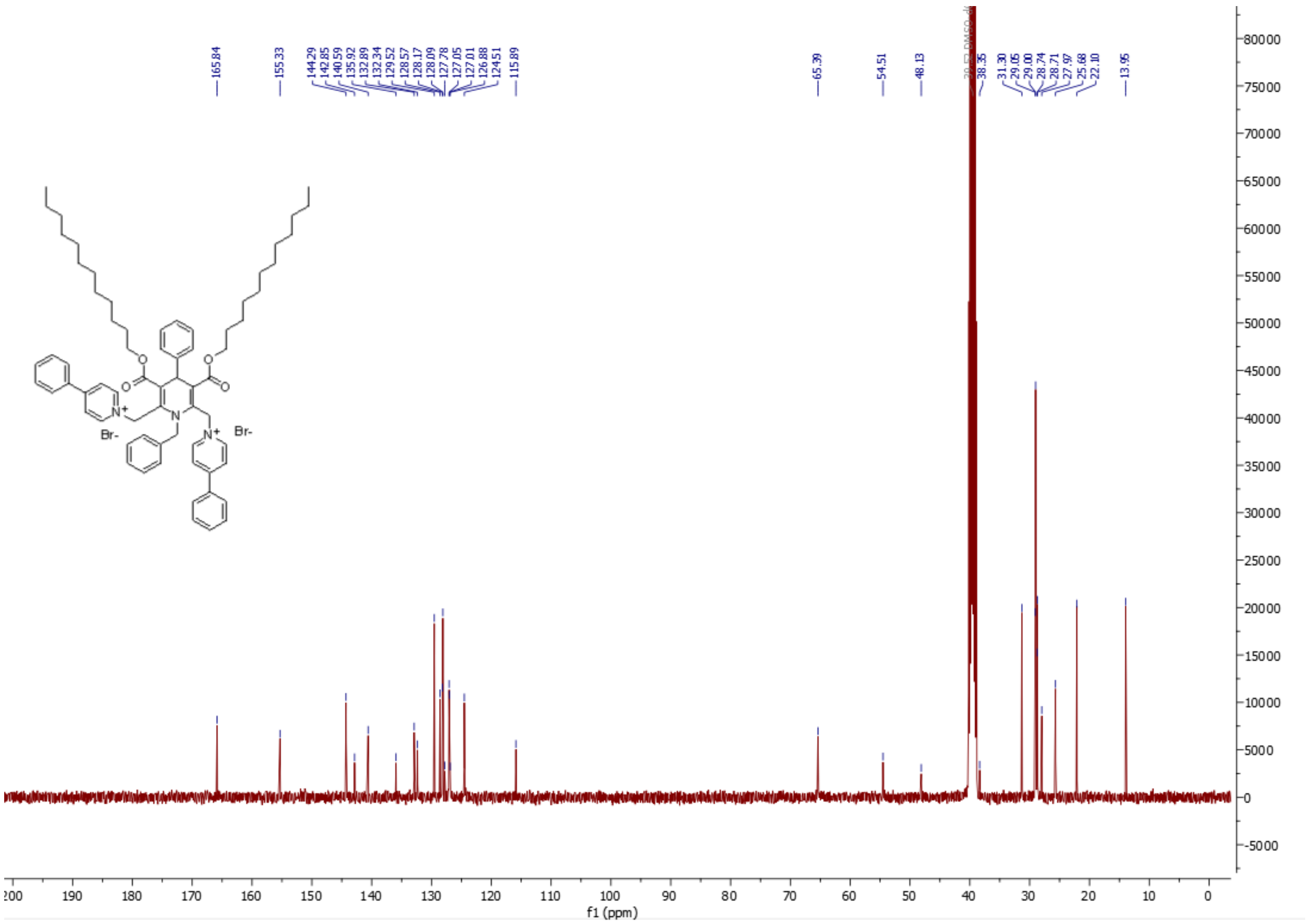

Figure S17. ^{13}C NMR spectrum of 1,1'-((1-benzyl-3,5-bis((dodecyloxy)carbonyl)-4-phenyl-1,4-dihydropyridine-2,6-diyl)bis(methylene))bis(4-phenylpyridin-1-ium) (**5d**)

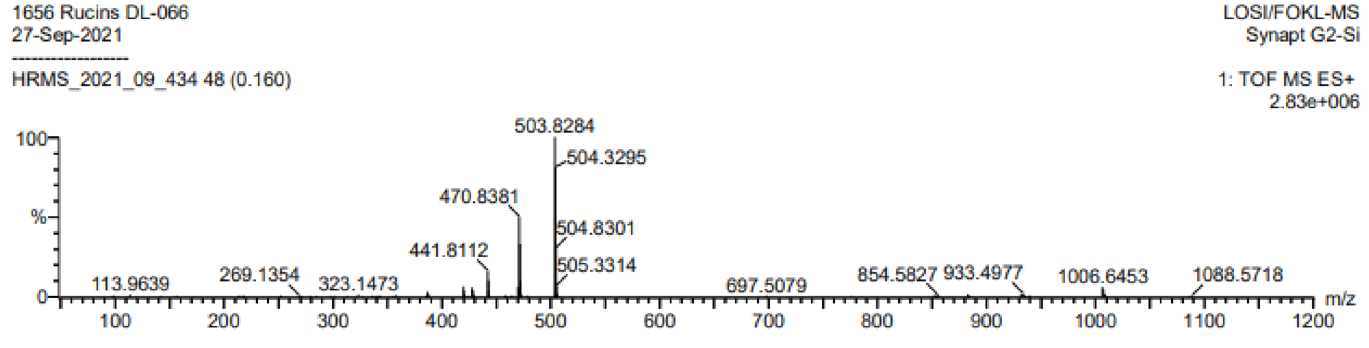

Minimum:				-1.5
Maximum:		3.0	10.0	50.0

Mass	Calc. Mass	mDa	PPM	DBE	Conf(%)	Formula
1006.6453	1006.6459	-0.6	-0.6	-1.5	0.00	C39 H92 N9 O20
	1006.6462	-0.9	-0.9	28.5	0.73	C68 H84 N3 O4
	1006.6440	1.3	1.3	11.5	0.00	C51 H88 N7 O13
	1006.6467	-1.4	-1.4	10.5	0.00	C55 H92 N O15
	1006.6435	1.8	1.8	29.5	99.27	C64 H80 N9 O2

Figure S18. HRMS data of 1,1'-((1-benzyl-3,5-bis((dodecyloxy)carbonyl)-4-phenyl-1,4-dihydropyridine-2,6-diyl)bis(methylene))bis(4-phenylpyridin-1-ium) (**5d**)

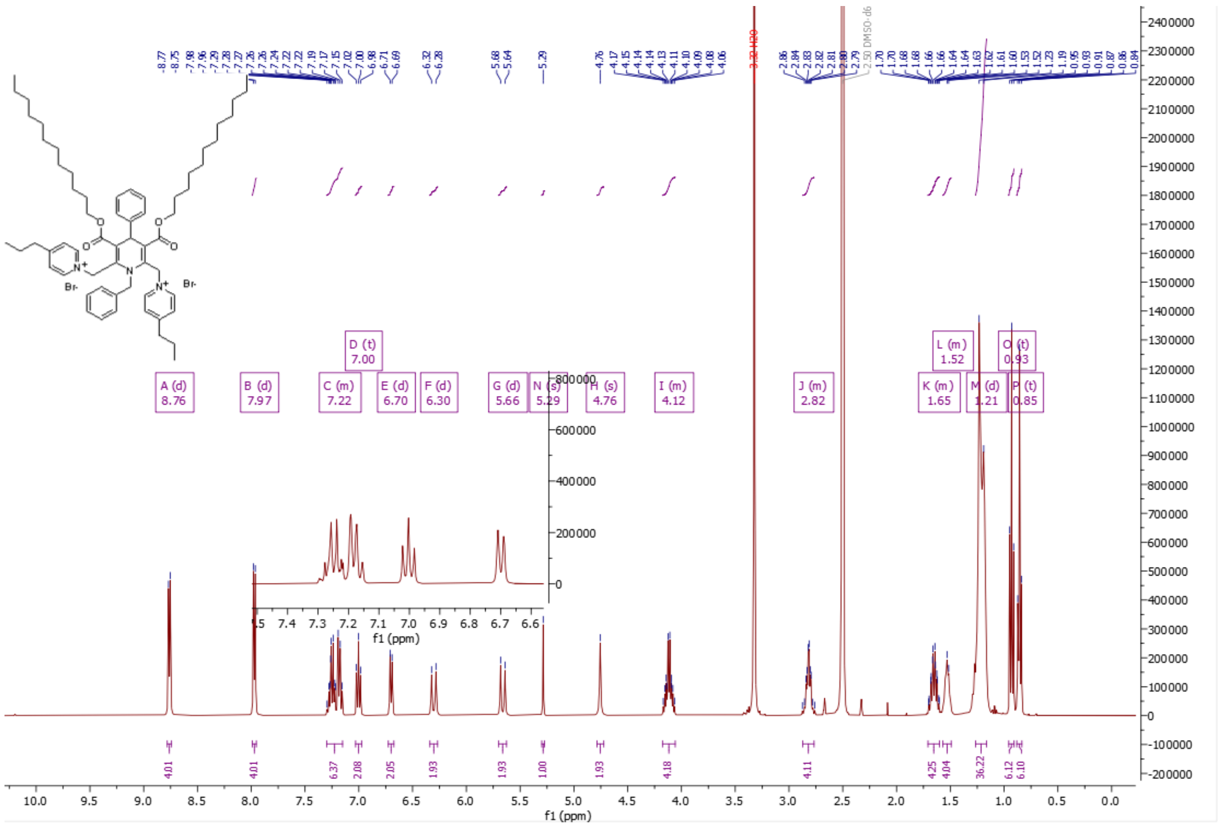

Figure S19. ^{1}H NMR spectrum of 1,1'-((1-benzyl-3,5-bis((dodecyloxy)carbonyl)-4-phenyl-1,4-dihydropyridine-2,6-diyl)bis(methylene))bis(4-propylpyridin-1-ium) (**5e**)

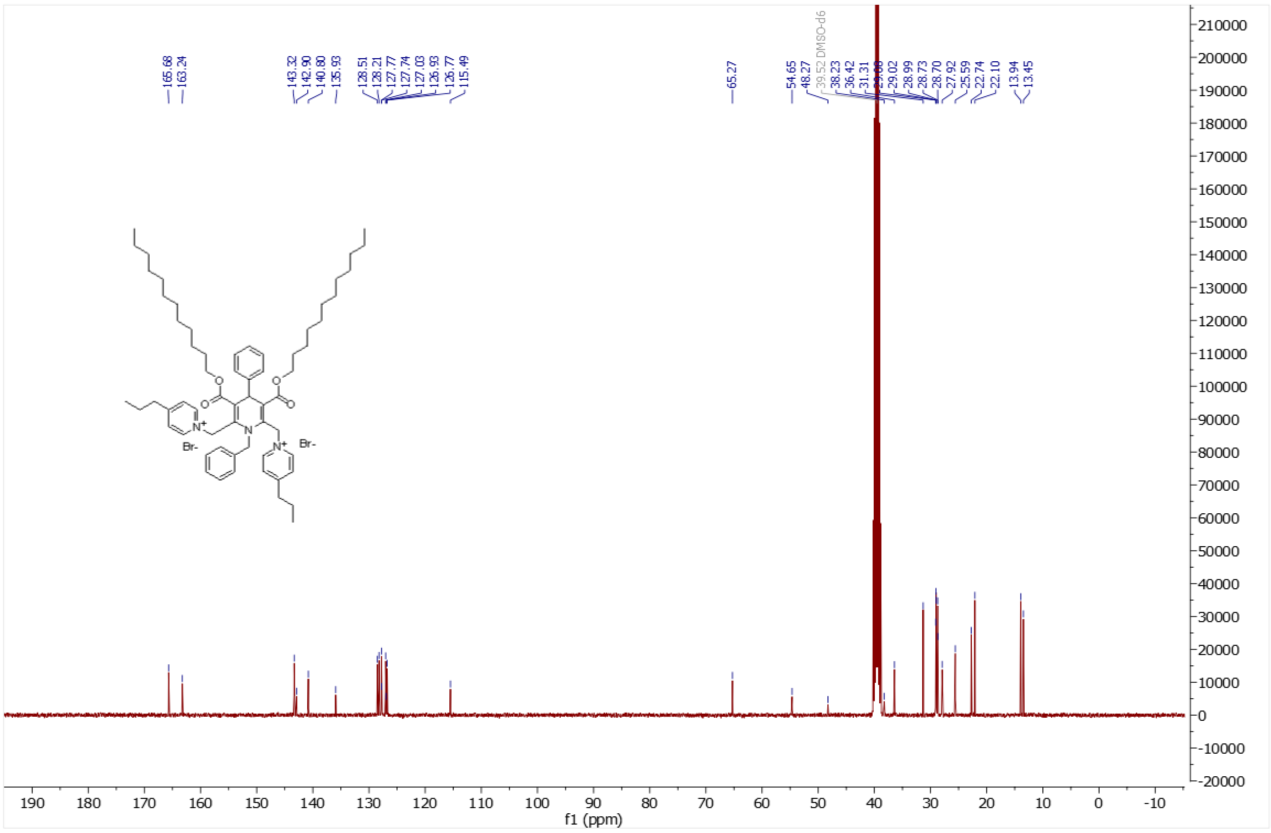

Figure S20. ^{13}C NMR spectrum of 1,1'-((1-benzyl-3,5-bis((dodecyloxy)carbonyl)-4-phenyl-1,4-dihydropyridine-2,6-diyl)bis(methylene))bis(4-propylpyridin-1-ium) (**5e**)

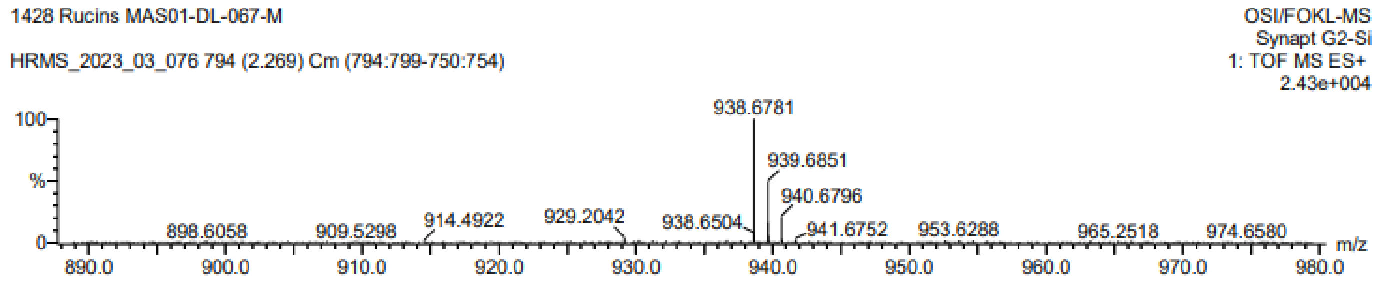

Minimum:	80.00				-0.5				
Maximum:	100.00		5.0	5.0	200.0				
Mass	RA	Calc. Mass	mDa	PPM	DBE	i-FIT	Norm	Conf(%)	Formula
938.6781	100.00	938.6775	0.6	0.6	20.5	488.1	1.487	22.60	C62 H88 N3 O4
		938.6788	-0.7	-0.7	25.5	488.4	1.803	16.48	C63 H84 N7
		938.6748	3.3	3.5	21.5	487.1	0.496	60.92	C58 H84 N9 O2

Figure S21. HRMS data of 1,1'-((1-benzyl-3,5-bis((dodecyloxy)carbonyl)-4-phenyl-1,4-dihydropyridine-2,6-diyl)bis(methylene))bis(4-propylpyridin-1-ium) (**5e**)

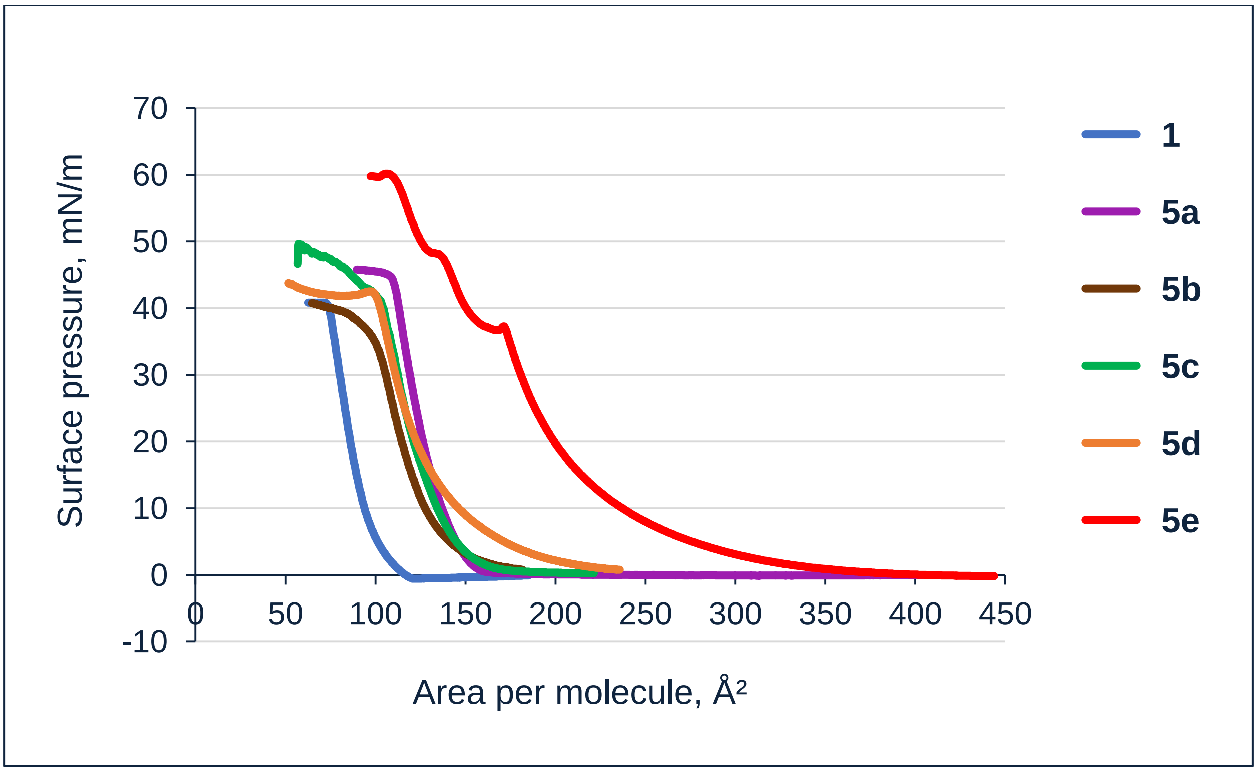

Figure S22. Surface pressure – mean molecular area isotherms of tested 1,4-DHP amphiphiles **1** and **5** at 25±1°C.

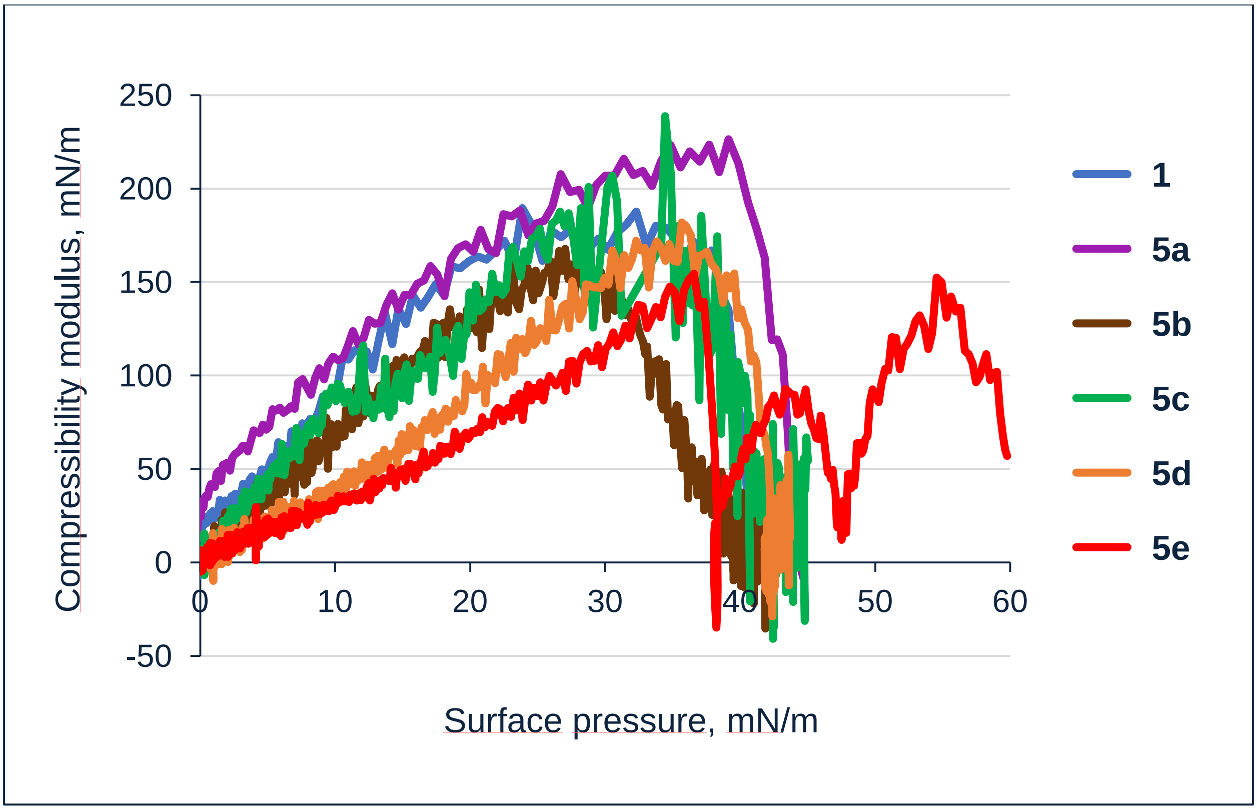

Figure S23. Compressibility modulus-surface pressure dependences obtained for the 1,4-DHP amphiphiles **1** and **5** monolayers.

Table S1. Values of Z-average ($Z_{av}D_H$) diameter and polydispersity index (PDI) of nanoparticles formed by 1,4-DHP amphiphiles **1** and **5** at the 0.1mM concentration obtained by DLS measurements for freshly prepared samples and after 1 month storage. The PDI value describes polydispersity of the sample; the Z_{av} diameter represents the average hydrodynamic diameter of all nanoparticles in the sample.

Sample concentration – 0.1 mM					
Entry	Comp.	Fresh samples		After 1 month storage	
		Z_{av} D_H, nm	PDI	Z_{av} D_H, nm	PDI
1	**1**	395 ± 28	0.342 ± 0.047	346 ± 3	0.305 ± 0.042
2	**5a**	781 ± 73	0.116 ± 0.112	873 ± 114	0.391 ± 0.027
3	**5b**	529 ± 258	0.521 ± 0.284	430 ± 44	0.443 ± 0.043
4	**5c**	715 ± 34	0.414 ± 0.087	458 ± 16	0.445 ± 0.02
5	**5d**	2572 ± 153	0.680 ± 0.328	1195 ± 148	0.731 ± 0.081
6	**5e**	462 ± 174	0.526 ± 0.251	1003 ± 566	0.782 ± 0.163

Table S2. Values of Z-average ($Z_{av}D_H$) diameter and polydispersity index (PDI) of lipoplexes formed by 1,4-DHP amphiphiles **1** and **5** and mRNA at different N/P charge ratios obtained by DLS measurements for freshly prepared samples. The PDI value describes polydispersity of the sample; the $Z_{av}D_H$ diameter represents the average hydrodynamic diameter of all nanoparticles in the sample.

Lipoplex size (fresh samples) at different N/P charge ratios							
Entry	Comp.	N/P = 1		N/P = 2		N/P = 5	
		Z_{av} D_H, nm	PDI	Z_{av} D_H, nm	PDI	Z_{av} D_H, nm	PDI
1	**1**	806 ± 60	0.246 ± 0.122	582 ± 90	0.127 ± 0.085	298 ± 49	0.266 ± 0.031
2	**5a**	1112 ± 169	0.48 ± 0.087	1325 ± 336	0.492 ± 0.083	143 ± 7	0.295 ± 0.071
3	**5b**	809 ± 88	0.349 ± 0.098	386 ± 9	0.42 ± 0.503	148 ± 7	0.179 ± 0.045
4	**5c**	1039 ± 215	0.357 ± 0.196	986 ± 75	0.514 ± 0.444	139 ± 2	0.215 ± 0.039
5	**5d**	2959 ± 2010	1 ± 0	1540 ± 198	0.373 ± 0.199	1551 ± 237	0.318 ± 0.233
6	**5e**	1535 ± 125	1 ± 0	1228 ± 266	0.439 ± 0.283	1208 ± 419	0.653 ± 0.305
